# A structured training program for health workers in intravenous treatment with fluids and antibiotics in nursing homes: A modified stepped-wedge cluster-randomised trial to reduce hospital admissions

**DOI:** 10.1371/journal.pone.0182619

**Published:** 2017-09-07

**Authors:** Maria Romøren, Svein Gjelstad, Morten Lindbæk

**Affiliations:** 1 Department of Administration Vestfold Hospital Trust, Tønsberg, Norway; 2 Department of General Practice Institute of Health and Society, University of Oslo, Blindern, Oslo, Norway; University of Glasgow, UNITED KINGDOM

## Abstract

**Objectives:**

Hospitalization is potentially detrimental to nursing home patients and resource demanding for the specialist health care. This study assessed if a brief training program in administrating intravenous fluids and antibiotics in nursing homes could reduce hospital transfers and ensure high quality care locally.

**Design:**

A pragmatic and modified cluster randomized stepped-wedge trial with randomization on nursing home level.

**Participants:**

330 cases in 296 nursing home residents from 30 nursing homes were included. Cases were patients provided intravenous antibiotics or intravenous fluids, in nursing home or hospital. Primary outcome was localization of treatment, secondary outcomes were number of days treated, days of hospitalization among admitted patients, type of antibiotics used and 30-day mortality.

**Intervention:**

The nursing homes sequentially received a one-day educational program for the health workers including theory and practical training in intravenous treatment of dehydration and infection, run by two skilled nurses. After completing the training program, the nursing homes had competence to provide intravenous treatment locally.

**Results:**

The intervention had a highly significant effect on treatment in nursing homes (OR 8.35, 2.08 to 33.6; P<0.01, or RR 2.23, 1.48 to 2.56). The number treated in nursing homes was stable over time; the number treated in hospital gradually decreased (chi square for trend P< 0.001).

Among patients receiving intravenous antibiotics in the nursing homes, 50 (46%) died within 30 days, compared to 30 (36%) treated in the hospital (P = 0.19). Among patients receiving intravenous fluids locally, 21 (19%) died within 30 days, compared to 2 (8%) in the hospital group (P = 0.34). Mortality was associated with reduced consciousness and elevated c-reactive protein.

**Conclusions:**

A brief educational program delivered to nursing home personnel was feasible and effective in reducing acute hospital admissions from nursing homes for treatment of dehydration and infections.

## Introduction

In the Norwegian population of 5.2 million inhabitants, there are 900 nursing homes and over 41 000 nursing home beds, and approximately 45% of all deaths occur here [1,2]. Nursing home residents are characterized by high age, frailty, chronic diseases and deficits in activities of daily living, and many have moderate to severe cognitive impairment, in Norway more than half [3–5]. Bacterial infections and dehydration contribute substantially to acute deterioration in nursing home residents, but treatment strategies and treatment goals is individual, multifactorial and context dependent [6]. Nursing home acquired infections has been a much studied topic, in particular the most common infections pneumonia and urine tract infections. Implementing diagnosis and treatment algorithms and guidelines for these conditions in long term care facilities have proved effective in improving quality of care; in some, but not all studies also with a reduction in hospital transfers [7–10].

Hospitalization from nursing homes is similarly complex; and transfer rates vary substantially between institutions and geographical areas [11, 12]. The need for intravenous treatment may be the only reason why many nursing home patients are transported to a hospital [13]. Hospitalization for acute care is considered potentially detrimental to the patient and resource demanding for the specialist health care [14]. Further high quality studies of interventions to reduce hospital admissions from nursing homes have been requested [11, 12].

As a response to these challenges, the local hospital and the Teaching Nursing Home in Vestfold county decided to conduct and evaluate an intervention to increase the competence in administrating intravenous fluids and antibiotics in all nursing homes in the county. The evaluation was designed as a pragmatic and modified stepped-wedge cluster randomised trial [15]. The number of nursing homes was high, making the stepped-wedge design with sequential rollout both feasible given the available resources, and reasonably efficient. The aim of the evaluation was to assess if a structured training program in administrating intravenous fluids and antibiotics on-site can reduce the number of hospital admissions among nursing home residents. Secondary outcomes presented are average length of treatment, 30-day mortality and number of days in hospital both before and after intervention; as well as these comparisons for treatment in nursing homes versus hospital; including appropriateness of antibiotic selection.

## Method

**Fig 1:**
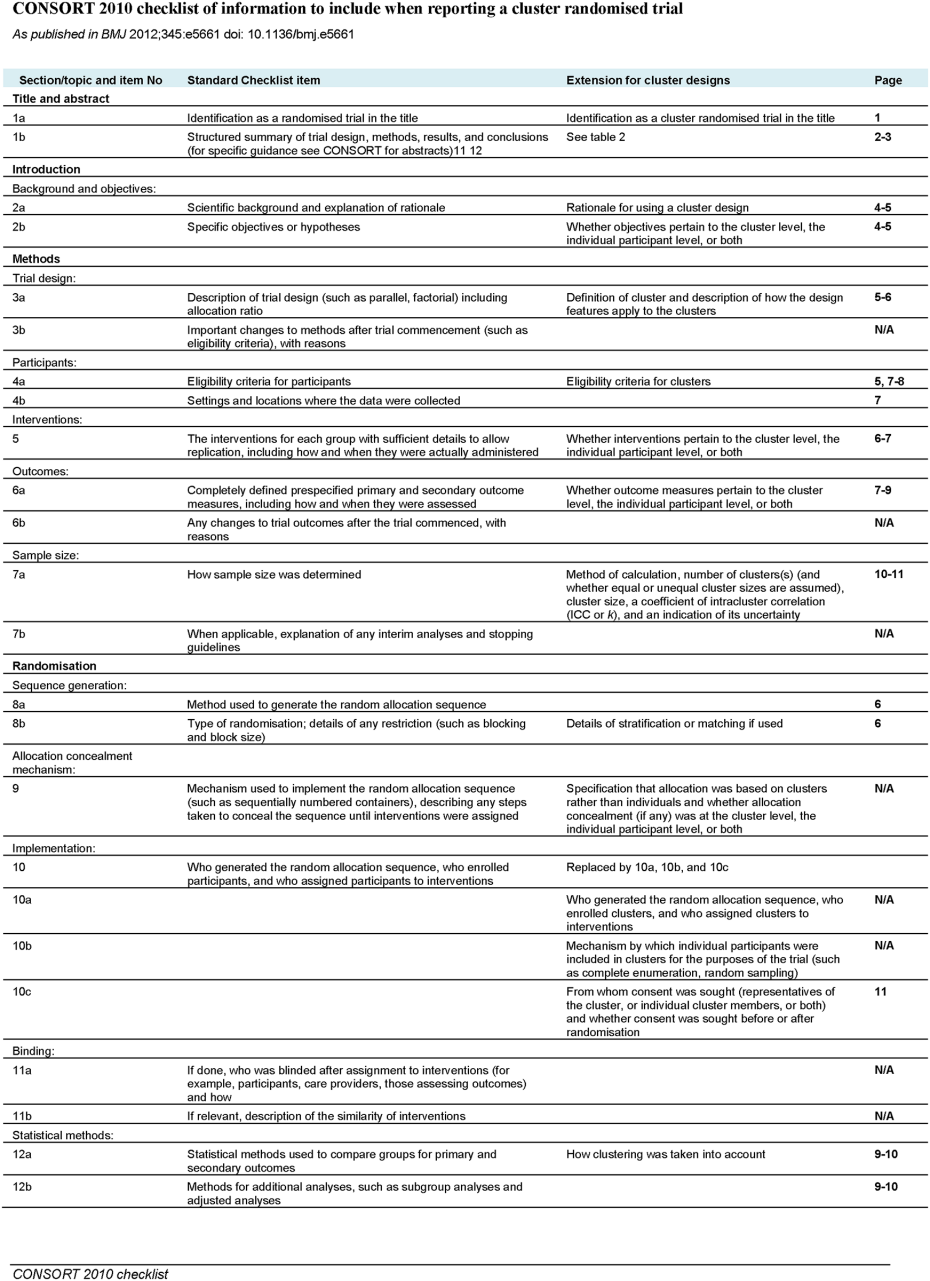
CONSORT 2010 checklist of information to include when reporting a cluster randomised trial. Suggested modifications to the CONSORT 2010 cluster extension for reporting of stepped wedge cluster randomised trials.

The study is reported in accordance with the Consort 2010 extension to cluster randomised trials and the suggested modifications to the Consort 2010 cluster extension for reporting of stepped wedge cluster randomised trials (Fig 1) [15]. Trial registration (12/1/09): ClinicalTrials.gov NCT01023763. The registration was delayed one month after study onset due to practical reasons. The authors confirm that all ongoing and related research within the trial is registered.

### Participants and setting

Eligible units were all 34 nursing homes in Vestfold County, Norway. Four declined to participate, two because the nursing home leaders perceived low need for intravenous treatment among their residents, two because they used the hospital in the neighboring county. The 30 participating nursing homes had 12–124 beds (median 41), in total 1379 beds. They had one to eight departments, and either one type of beds or a combination of beds: for rehabilitation, short term and long term care, palliative care and special departments for patients with dementia. Mean man-years for nurses in the nursing homes was 14.1 (range 3.5–40.2), mean man-years for nursing assistants were 26.2 (range 5 to 105).

We used 50 beds as a cut off and defined nine nursing homes as large, 21 as small. Two of the large nursing homes received the intervention as a pilot project to assess the training material, equipment etc. They were not randomized and did not serve as controls pre-intervention. These two and three other nursing homes had a certain competence and routine in administrating intravenous treatment before the project started, such as in the palliative units.

There is one hospital in the county: a local public hospital, Vestfold Hospital Trust. All nursing home patients in need of hospitalization are admitted to this hospital, and all admissions in this study were to the Medical department.

### Trial design and randomization

We conducted a pragmatic and modified stepped wedge cluster randomized trial with randomization on the nursing home level, each nursing home representing one cluster. The design involves random and sequential crossover of clusters from control to intervention until all clusters are exposed. Data collection continues throughout the study so that each cluster contributes observations under both control and intervention observation periods [15, 16]. We selected a stepped wedge design in order to retain the power of randomization while offering all facilities enrolled in the trial exposure to what was expressed to be a desirable intervention and to enable delivery of the intervention to these facilities by a small study team. The modification refers to including the pilot sites in the intention to treat-analysis.

**Fig 2:**
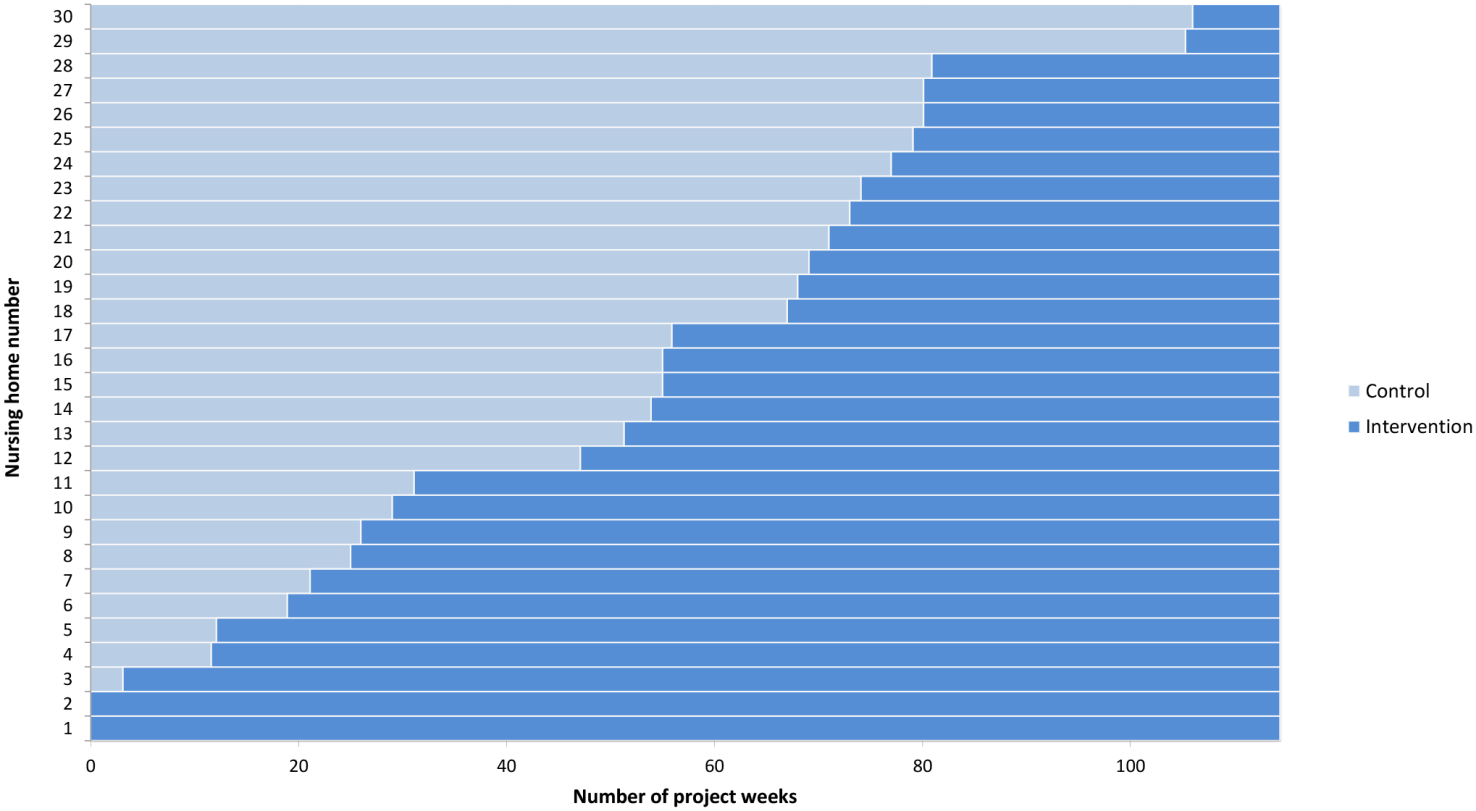
Modified stepped-wedge design with 30 clusters (nursing homes). Each cluster receive the intervention at baseline. The median length of the steps (intervals between each crossover) was 14 days (0–171 days).

The formal trial period was from 1^st^ of November 2009 to the 31^th^ of December 2011. The intervention was implemented in the 30 nursing homes in accordance to the randomization plan from 11^th^ of November 2009 to 1^st^ of November 2011, resulting in a random and sequential crossover of clusters from control group pre-intervention to intervention group after implementation (Fig 2). The patient-level inclusion and data collection continued during the same period (first patient was included 17^th^ of November 2009, last patient included 19^th^ of December 2011) so that each nursing home except the pilot nursing homes potentially could contribute with cases under both control and intervention periods.

The randomization was stratified based on nursing home size and followed two computer based lists, one with the seven large and one with the 21 small nursing homes. In order to get a balanced randomization, we randomised three small nursing homes and then one large nursing home consecutively. The date of inclusion of the pilot sites was defined as onset of the study (day 0). The intention was to include the remaining nursing homes one by one, giving 29 steps. The randomization list was open to the intervention team, and the two nurses who ran the training program cooperated so that each of them included every second nursing home consecutively according to the list. In two instances, they made appointments with their respective nursing homes on the same day, resulting in two sites being transitioned simultaneously (step 13 and 23).

The study was not designed to have a fixed time between the steps. The intervention was carried out in ordinary nursing homes with normal operation and activity, and the time to the next step was determined by when it was feasible for each nursing home to receive two ore more days of education within the frames of day-to-day care. For example, the educational program could not be run during holidays with less staff and few of the permanent employees on duty.

The median length of the steps was 14 days (0–171 days). The trial design is presented in Fig 2.

### Intervention and training

The intervention was a structured educational program in intravenous treatment of dehydration and infections for all health workers in the nursing homes (registered and enrolled nurses and nurse assistants with and without formal education). Two nurses from the Vestfold Hospital Trust ran the training program simultaneously in half of the nursing homes each. They did not receive specific training for the purpose, but both had solid experience in intravenous treatment to elderly. The exact timing of the training, also representing the switch from control to intervention period, and subsequently the length of time between each nursing home switch, was determined by the capacity of the nurses and the specific nursing home.

The training lasted one day, was held in the nursing home, and included theory of prevention, presentation, diagnosis and treatment of dehydration and infections (based on PowerPoint presentations) and practical training in peripheral intravenous therapy skills and procedures (using intravenous training arms). It was repeated one to three times in each site, to ensure participation for all relevant personnel. The number of nursing staff trained was not registered systematically, but the nursing home- and ward managers reported that all or the majority of their employees participated. The few nurses or nursing assistants who did not manage to participate (mainly due to part-time contracts and shift work which is very common in Norwegian nursing homes) were offered to visit the Simulation Centre at the hospital for practical training. Two of the researchers contacted the nursing homes monthly for assistance and support regarding treatment or data collection in the study period and were in addition available for questions on a daily basis.

In nursing homes that had completed the training program (intervention period), and had sufficient expertise and capacity, patients in need of intravenous fluids or antibiotics were treated locally; otherwise they were hospitalized. The control group received “standard practice”, i.e. patients were hospitalized by the nursing home doctor for intravenous treatment. As described, a few of the larger nursing homes provided intravenous treatment before the project started, explaining why a number of patients were treated locally in the control period.

### Recruitment and data collection

Inclusion of patients: A case was defined as a patient provided intravenous treatment in either nursing homes or hospital. We defined two groups: 1. Patients provided *intravenous antibiotics* for pneumonia, urinary tract infection or skin infection, with or without additional intravenous fluids, and 2. Patients provided *intravenous fluids*: in conjugation with an infection (with or without oral antibiotics); due to reduced intake of fluids; due to hypotension; as a part of terminal care etc. Inclusion criteria for patients admitted to the hospital was that they could have been diagnosed and treated at the nursing home given necessary competence and available personnel and equipment. Patients with septicemia and patients in need of hospitalization for additional diagnostics or treatment, were not included in the study.

Demographic and clinical data collected is listed in Table 1. Demographic data were age, gender, co-existing diseases and Barthel Index of Activities of Daily Living 14 days before disease onset Clinical data were recorded in 30 days: at enrollment (day 1 in the treatment course) and at given days during the course of the acute illness: diagnosis, vital signs (blood pressure, pulse, temperature, respiratory rate), c-reactive protein (CRP) value, food and fluid intake, consciousness, delirium assessed with Confusion Assessment Method (CAM).Direct and indirect complications related to the acute disease as well as type of intravenous fluids or antibiotics were also registered.

**Table 1:** Characteristics of patients provided intravenous antibiotics or fluids in nursing homes and hospital, by control and intervention group. Values are numbers (percentages) unless stated otherwise. Calculation of p-values was done by independent samples T-test (two-sided) for comparing means, and two-sided chi-square test for comparing differences in counts.

		Control			Intervention			Total	P-values	
		Nursing home (n = 38)	Hospital (n = 64)	Total (n = 102)	Nursing home (n = 184)	Hospital (n = 44)	Total (n = 228)	n = 330	Control vs. intervention	Nursing home vs.hospital
**Mean age (range)**		79,6 (52–95)	81,8 (38–98)	81,0 (38–98)	81,0 (45–99)	83,9 (71–93)	81,6 (45–99)	81,4	0.02	0.11
**Median age**		81.0	85.0	84.0	83.0	85.5	84.0	84.0	0.02	0.11
**Women**		28 (73)	41 (64)	69 (69)	98 (53)	21 (48)	119 (52)	188 (57)	<**0,01**	0.91
**Barthel Index of ADL** (n = 74/133)	Mean Median Range	6,2 6.0 (0–20)			6,6 5.0 (0–20)			6.5 5.0 (0–20)	0.72	N/A
**Number regular medications**	**(mean)**	7,9	8,4	8,2	8,6	9,0	8,7	8,5	0.31	0.80
**Co-existing diseases**										
Apoplexia (n = 271)		5 (32)	20 (33)	25 (32)	32 (22)	12 (27)	44(23)	69 (26)	0.11	0.18
COPD (n = 271)		3 (21)	16 (25)	19 (24)	28 (19)	14 (33)	42 (22)	61 (23)	0.59	0.08
Angina pectoris (n = 270)		7 (50)	17 (27)	24 (31)	33 (22)	18 (41)	51 (26)	75 (28)	0.43	0.14
Heart failure (n = 270)		4 (29)	22 (35)	26 (34)	35 (24)	27 (61)	62 (32)	88 (33)	0.80	<**0.01**
Diabetes (n = 271)		1 (7)	15 (24)	16 (21)	21 (14)	7 (16)	28 (14)	44 (16)	0.20	0.12
Cancer (n = 270)		3 (21)	10 (16)	13 (17)	62 (42)	12 (27)	74 (38)	87 (32)	<**0,01**	<**0.01**
**Diagnosis in patients treated with i.v. antibiotics**										
Pneumonia		13 (65)	26 (54)	39 (57)	63 (70)	21 (58)	84 (67)	123 (63)	0.20	0.06
Pneumonia & urinary tract infect.		3 (15)	13 (27)	16 (24)	7 (8)	8 (22)	15 (12)	31 (16)	**0.04**	<**0.01**
Upper urinary tract infection		3 (15)	7 (15)	10 (15)	10 (11)	6 (17)	16 (13)	26 (13)	0.70	0.46
Other infections*		1 (5)	2 (4)	3 (4)	10 (11)	1 (3)	11 (9)	14 (7)	0.27	0.09
**Diagnosis in patients treated with i.v. fluids**										
Infection (with/wthout registered reduced intake, hypotension etc)		13 (72)	10 (63)	23 (68)	66 (70)	6 (75)	72 (71)	95 (70)	0.75	0.71
No infection (reduced intake, hypotension etc.)		5 (28)	6 (38)	11 (32)	28 (30)	2 (25)	30 (29)	41 (30)	-	-
**Clinical status on enrollment (day 1)**										
Systolic BP (mean/median)		120/109	138/130	132/125	122/120	140/137	127/124	125/123	0.06	<**0.01**
Pulse (mean/median)		92/99	93/88	93/88	84/86	90/85	86/85	87/85	0.30	**0.05**
Respiratory rate (mean/median)		18/18	22/22	21/20	21/20	23/20	21/20	21/20	0.72	0.24
Temp (mean/median)		37.5/37.2	37.6/37.7	37.6/37.6	37.5/37.2	37.3/37.3	37.5/37.3	37.5/37.4	0.46	0.31
Septicemia score > 2		1 (3)	4 (6)	5 (5)	7 (4)	3 (7)	10 (4)	15(5)	0.84	0.24
Reduced consciousness		22 (58)	18 (28)	40 (39)	83 (45)	15 (43)	98 (43)	138 (42)	0.52	<**0.01**
CRP-value (mean/median)		79/63	132/132	116/124	108/97	152/178	119/101	111/101	0.55	<**0.01**
Reduced food intake		35 (92)	54 (84)	89 (87)	164 (89)	39 (89)	203 (89)	292 (89)	0.64	0.35
Reduced fluid intake		34 (90)	57 (89)	91 (89)	165 (90)	39 (89)	204 (90)	295 (89)	0.94	0.84
Delirium		4 (11)	15 (23)	19 (19)	27 (15)	7 (16)	34 (15)	53 (16)	0.43	0.17

*Skin, gastrointestinal and unspecified infections

In each of the nursing homes as well as in each hospital department, one or several nurses served as primary contact (PC) for the study team. These were responsible for including and registering information about the patients receiving intravenous treatment in standardized data collection forms. The nursing homes were followed closely by the study team, both regarding the local intravenous treatment and the patient inclusion and data collection. The PCs were contacted for a follow-up by telephone on a regular basis. In addition, the study team was available for support to the nursing homes and on e-mail and telephone on a daily basis. The nursing homes received a follow-up visit some months after the intervention, a few were visited several times. Complete patient inclusion was easier to control at the hospital. Twice weekly, a list of admissions to the Medical Department was provided, and the study team ensured inclusion of all patients filling the inclusion criteria.

### Outcome measures

Outcomes were measured in individual residents. The unit of analysis was the treatment level, whereas the cluster level was the nursing homes, serving as the unit of allocation and intervention. The primary outcome measure was number of patients treated with intravenous antibiotics and/or fluids in a participating nursing home or in Vestfold Hospital Trust. Secondary outcome measures were number of days treated, number of days of hospitalization among the admitted patients, type of antibiotics used, and mortality within 30 days. The antibiotic selection was compared with the national guidelines for antibiotic use in nursing homes and in hospital [17, 18]. Information on other health care related treatment outcomes collected will be reported elsewhere.

### Statistical analysis

IBM SPSS^®^ statistics program and STATA 12 were used for statistical analyses. The logistic regression analyses were performed as multilevel models with nursing homes as clusters (random intercept). Comparison of means were analysed by independent samples T-test (two-sided alpha). The Stata function "CLTEST" was used to perform cluster-adjusted Chi-square tests (P-value from the group adjustment Chi-2) for comparing differences in counts. All analyses were conducted on an intention-to-treat-basis. The nursing homes in the pilot study were included in the analyses to increase the sample size. Identical analyses were also performed without the two pilot sites. In all the logistic regression analyses, the identity of the nursing home was used as the cluster identification (random intercept). In the bivariate and multivariate logistic regression analysis, the dependent outcome variable described whether the patient was treated in a nursing home or in the hospital. The associations are presented in odds ratios (OR) and for the main outcome an estimated relative risk (RR) [19]. Independent variables were age, gender, number of regular drugs, Barthel Index and the co-existing diseases and the measures of clinical status on day 1 listed in Table 1, intravenous fluids or antibiotics provided and intervention or control period. Variables not significantly associated with location of treatment in bivariate analysis were not included in multivariate analysis (except gender). The variables CRP and blood pressure (BP) were grouped into tertiles; the level of consciousness was dichotomized to “awake” or “reduced consciousness or somnolence”. To assess comparability between patient groups, we used the chronic diseases recorded in the collection forms, and the number of regular medications, as a proxy for the patients’ general health status.

The time variation variable, describing how many nursing homes that were included at the time of each patient event, was recoded into a six-category variable, as the original variable had 30 different categories that would make the resulting results more difficult to interpret.

We used a significance level of p < 0.05 for all analyses. Explanatory variables with a value of p > 0.2 in a bivariate multilevel regression were excluded from the multivariate model, with exception of gender. In the multilevel logistic regression models we used nursing home as the cluster level, and calculated the intra cluster correlation coefficient (ICC), using the STATA function “estat icc”.

Few patients were included more than once: 273 patients (92%) were included once,19 patients (6.4%) twice, 2 patients (1%) three times, 1 patient (0.3%) 6 times and 1 patient (0.3%) 7 times. Allowance for repeated measures on individuals was therefore not included in the analysis.

### Sample size estimation

There was no previous research on nursing home patients in need of hospitalization for intravenous treatment in this setting. The power calculation was based on assumptions and discussions with health workers and administrators in the field. As some nursing homes already provided intravenous treatment, we estimated that 10% of the patients were treated locally at baseline. We further estimated a 25% reduction of hospital admissions of patients in need of intravenous treatment, from 90% to 65%. We used a two-sided alpha level of 0.05 and a beta of 0.80. We assumed a cluster-coefficient of 0.10. The calculation gave an estimate of 56 patients in each group. With a calculated drop out proportion of 10%, the estimated number of patients needed to treat increased to 65 patients in each group, totally 130 patients, or 4.3 patients from each of the 30 nursing homes. The original power calculation was for a standard RCT, allowance for the number of steps and allowance for any repeated measures on individuals was not included in this sample size calculation.

### Patient involvement

Patients were not involved in the design, development of outcome measures, recruitment to or conduct of the study, but patients’ priorities, experiences, and preferences was indirectly taken into account. The Teaching Nursing Home played an important role in planning and implementation of the project, and nurses from all nursing homes were involved in planning the study, recruitment of patients and collection of data. The results is planned to be disseminated to the participating nursing homes and the hospital through seminars and workshops for the health personnel and administrators. We also aim to make the results known to lay people through mass media.

### Ethical considerations

The Regional Committee for Medical Research Ethics verbally communicated the approval of the collaborative research project after a committee meeting 19^th^ October 2009, confirmed by letter the 13^th^ November 2009 (reference no. 2009/1584a-1). Their assessment of the burden of the intervention on patients concluded that the intervention was beneficial to nursing home patients. Written informed consent was obtained from all patients. In patients lacking decision-making capacity, written consent was collected from next of kin.

## Results

### Numbers analyzed

**Fig 3:**
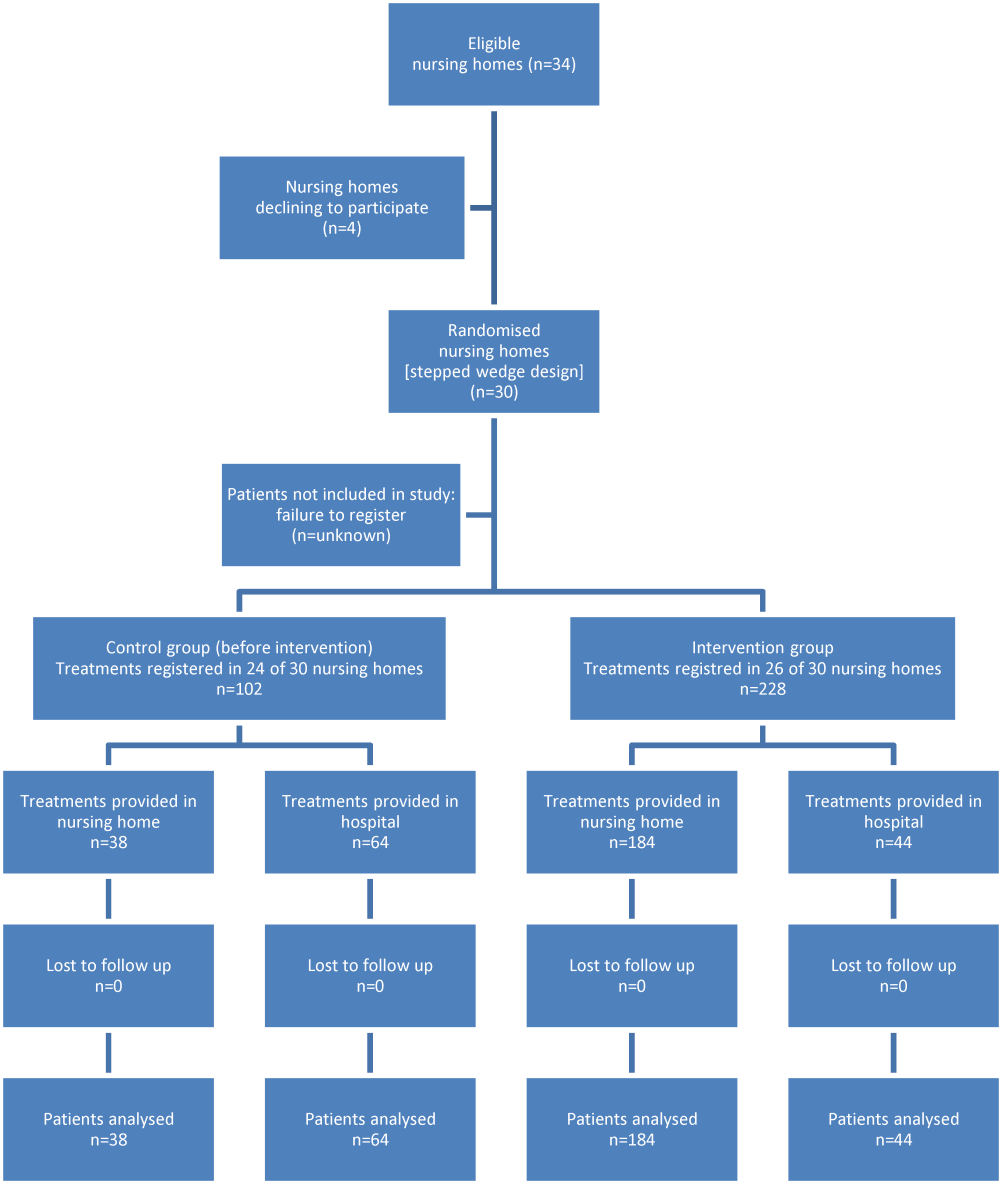
Flowchart showing nursing home and patient recruitment. Patients that did not fill the inclusion criteria, or patients who by mistake were eligible, but not included, were not registered. All eligible patients consented to participate and no patients were excluded. None of the included patients were lost during the 30 days follow up; death in the period was regarded as an outcome.

296 patients with 330 treatments were included during the 26-month period; Fig 3 displays the participant flow for the study.

Table 2 gives the number of patients treated locally or admitted to hospital in each nursing home before and after intervention. Intention to treat analysis was conducted for the 2 pilots and the 28 nursing homes randomized into the intervention. The two pilots and four additional nursing homes had no intravenous treatments registered in the control period; four nursing homes had no treatments registered after the intervention.

**Table 2:** Number of patients provided intravenous treatments in nursing home or hospital and treatments per 100 beds per month in each of the 30 nursing homes in Vestfold, Norway in the study period 2009–2011.

		Control period					Intervention period					Total
		Hospital		Nursing home		Treatmentsper 100beds/month	Hospital		Nursing home		Treatments per 100beds/month	
Nursing home no.	Number of beds	No. iv ab	No. iv fluids	No. iv ab	No. iv fluids		No. iv ab	No. iv fluids	No. iv ab	No. iv fluids		
**1**	124	0	0	0	0	-	3	1	14	11	0.90	29
**2**	122	0	0	0	0	**-**	5	1	42	40	2.77	88
**3**	19	0	0	0	0	0	1	0	0	6	1.42	7
**4**	22	0	0	0	0	0	0	0	1	3	0.76	4
**5**	48	0	0	0	0	0	3	0	4	5	1.04	12
**6**	38	0	0	1	0	0.66	1	0	1	4	0.72	7
**7**	12	1	0	0	0	2.08	1	0	0	1	0.76	3
**8**	68	1	0	0	0	0.29	5	0	0	3	0.56	9
**9**	26	5	0	0	1	4.62	3	1	4	1	1.65	15
**10**	16	1	0	0	0	1.04	1	0	0	1	0.63	3
**11**	69	2	2	3	0	1.69	2	0	3	2	0.51	14
**12**	28	3	1	0	1	1.79	1	0	1	1	0.67	8
**13**	20	0	0	0	1	0.45	0	1	0	0	0.33	2
**14**	16	3	0	0	0	1.56	0	1	4	1	2.68	9
**15**	86	1	0	2	1	0.39	4	0	1	1	0.50	10
**16**	18	0	0	0	2	0.93	0	0	0	1	0.40	3
**17**	32	2	2	0	1	1.30	0	0	8	1	2.01	14
**18**	58	3	0	0	0	0.34	1	0	0	4	0.78	8
**19**	64	0	0	0	0	0	0	0	1	0	0.14	1
**20**	52	4	5	0	0	1.15	0	0	0	0	0	9
**21**	33	0	0	1	0	0.19	1	0	1	0	0.61	3
**22**	20	4	0	1	2	2.19	1	2	0	2	2.50	12
**23**	76	3	1	0	0	0.33	1	0	0	1	0.26	6
**24**	25	2	1	0	0	0.71	1	0	0	0	0.44	4
**25**	48	0	1	2	0	0.37	0	0	0	0	0	3
**26**	56	3	0	10	3	1.59	0	1	5	3	2.01	25
**27**	46	2	2	0	0	0.48	1	0	0	1	0.54	6
**28**	38	2	1	0	0	0.44	0	0	0	0	0	3
**29**	55	4	0	0	0	0.32	0	0	0	1	0.61	5
**30**	44	2	0	0	6	0.76	0	0	0	0	0	8
**Sum**	1 379	48	16	20	18	0.86	36	8	90	94	0.87	330

Despite tight follow-up by the research team, we discovered that not all patients treated locally were included in the study. Reasons given by the PCs for not including patients were mainly lack of time or lack of dedication among the staff to adhere to the data collection. We do not know the exact number of patients that were treated in the nursing homes or if the non-inclusion varied throughout the period. We have no reason to believe that patients not included in the study differed from patients included.

### Participant characteristics

Table 1 displays participant characteristics at the time of inclusion. The patients in the control and intervention group were similar in most of the characteristics except a higher proportion of women in the control group; a higher proportion of patients with cancer in the intervention group and a higher proportion of combined pneumonia and UTI in the control group. Among patients treated with intravenous antibiotics, pneumonia was the dominating diagnosis: 57% (95% confidence interval 45 to 69%) before and 67% (58 to 75%) after the intervention, P = 0.12). Among patients treated with intravenous fluids before the intervention, 23 (68%, 51 to 84%) had an infection and 11 (48%, 26 to 70%) were treated with oral antibiotics; after the intervention, 72 (71%, 62 to 80%) had an infection (P = 0.746), and 21 (57%, 45 to 69%) were treated with oral antibiotics (P = 0.44).

Comparability between patients treated locally and patients admitted to hospital is necessary for the comparison of clinical outcome in the two treatment levels. The major difference was that 110 of 222 (50%, 43 to 56%) of the patients treated in the nursing home and 84 of 108 (78%, 70 to 86%) patients treated in the hospital were provided intravenous antibiotics (P<0.001). Further, among patients receiving intravenous antibiotics, 21 (25%, 16 to 34%) had a combined pneumonia and urinary tract infection in the hospital versus 10 (9%, 4 to 15%) in the nursing homes (P<0.001). The proportion of patients with heart failure was lower in the nursing home group than in the hospital group (24%, 17 to 31% versus 46%, 36 to 55%, P<0.001), while cancer was more frequent (40%, 32 to 47% versus 21%, 13 to 28%, P<0.001). Of the vital signs on treatment day 1, the systolic blood pressure was lower in the nursing home group (mean 123 mmHg, 118 to 127 mmHg, versus 135 mmHg, 129 to 141, P<0.001); the pulse was lower (mean 85 (82 to 88) versus 90 (86 to 94), P<0.001); a higher proportion had a reduced level of consciousness (47% (41 to 54%) versus 31% (22 to 39%), P<0.01) and CRP was lower (mean 100 (88 to 112) versus 130 (113 to 148), P<0.001).

### Primary outcome: Location of intravenous treatment

In the majority of the nursing homes, few patients received intravenous treatment—regardless of location: median 0.47 patients were treated per 100 beds per month (range 0–4.6) before the intervention and median 0.62 patients were treated per 100 beds per month (range 0–2.8) after the intervention (Table 2). The proportion of patients treated in the nursing home increased from 37% (28 to 47%) in the control period to 81% (76 to 86%) in the intervention period (P<0.05) (Table 3). The proportion of patients treated with intravenous fluids in the nursing homes increased from 53% (35 to 71%) to 92% (87 to 97%), P<0.001, whereas the proportion of patients treated with intravenous antibiotics in the nursing homes increased from 29% (18 to 41%) to 71% (63 to 79%), P<0.001. The two pilot nursing homes had the highest number of patients treated locally. When we excluded these two nursing homes, the proportion of patients treated locally with intravenous fluids increased from 53% (35 to 71%) to 88% (78 to 97%) after intervention (P<0.001), and the proportion of patients treated with intravenous antibiotics increased from 29% (18 to 41%) to 55% (42 to 68%) (P<0.005).

**Table 3:** Number of patients receiving intravenous antibiotics and fluids in hospital versus nursing home in the intervention and control group. Values are numbers (percentages). The calculated p-values are adjusted for clustering at the nursing home level.

	Control		Intervention		P-values
	n	(%)	n	(%)	
**Patient provided antibiotics**					
Nursing home	20	(29)	90	(71)	
Hospital	48	(71)	36	(29)	
Total	68	(100)	126	(100)	<0.05
**Patients provided intravenous fluids**					
Nursing home	18	(53)	94	(92)	
Hospital	16	(47)	8	(8)	
Total	34	(100)	102	(100)	<0.05
**All patients treated**					
Nursing	38	(37)	184	(81)	
Hospital	64	(63)	44	(19)	
Total	102	(100)	228	(100)	<0.05

**Fig 4:**
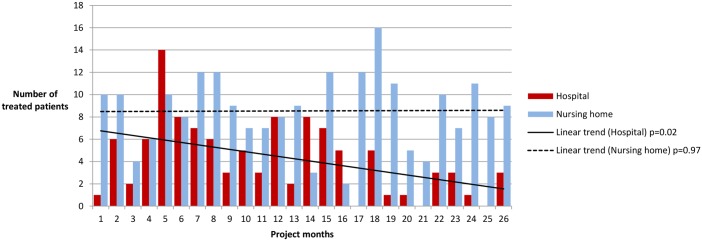
Number of patients receiving intravenous antibiotics and fluids in nursing home versus hospital in the study period, per 3 months.

Fig 4 shows the development of number and location of iv-treatments over time. The number treated in nursing homes is stable over time (linear trend -0.04, P = 0.97), while the number treated in hospital gradually reduced through the project period (linear trend -2.38, P = 0.02). The difference between these two groups is significant (chi square for trend P< 0.001). We found a similar trend without the pilot sites included (S1 Fig)

The multivariate analysis adjusting for covariates confirmed that there was a highly significant effect of the intervention on treatment in nursing homes (OR 8.35 (2.08 to 33.6), P<0.01, corresponding to RR 2.23, 1.48 to 2.56 (Table 4). Congestive heart failure and the clinical variables high blood pressure and CRP in the upper tertile associated significantly with admission to hospital. The nursing home group level ICC was estimated to 0.51 (0.26 to 0.76). The results were similar without the pilots included (S1 Table).

**Table 4:** Associations of demographic and clinical variables with intravenous treatment in the nursing home. Multilevel logistic regression model with nursing home as cluster (random intercept).

Factors	Bivariate analysis			Multivariate analysis (N = 249)		
	OR	(95% CI)	P-value	OR	(95% CI)	P-value
**Intervention**	5.60	2.79 to 11.2	<0.01	8.35	2.08 to 33.6	<0.01
**Intravenous antibiotics**	0.18	0.09 to 0.36	<0.01	0.89	0.31 to 2.54	0.82
**Gender**	0.69	0.38 to 1.23	0.21	0.95	0.39 to 2.29	0.90
**Reduced consciousness**	3.21	1.71 to 6.05	<0.01	2.12	0.84 to 5.38	0.11
**Systolic blood pressure at onset (tertiles)**						
<115 mmHg	Reference			Reference		
115 to 138 mmHg	0.65	0.31 to 1.37	0.26	0.84	0.27 to 2.56	0.75
>138 mmHg	0.33	0.16 to 0.67	<0.01	0.33	0.11 to 2.56	0.04
**CRP at onset (tertiles)**						
<65	Reference			Reference		
65 to 156	0.78	0.36 to 1.67	0.52	0.75	0.24 to 2.33	0.62
>156	0.25	0.11 to 0.57	<0.01	0.20	0.06 to 0.73	0.02
**Congestive heart failure**	0.20	0.09 to 0.42	<0.01	0.15	0.06 to 0.42	<0.01
**Number of nursing homes in intervention (time factor)**						
1 to 5	Reference			Reference		
6 to 10	0.57	0.21 to 1.54	0.27	0.67	0.11 to 3.94	0.66
11 to 15	1.30	0.49 to 3.43	0.60	1.74	0.31 to 9.73	0.53
16 to 20	0.45	0.15 to 1.29	0.14	0.53	0.09 to 3.05	0.48
21 to 25	4.46	1.11 to 17.9	0.04	6.59	0.58 to 75.5	0.13
26 to 30	3.83	1.27 to 11.5	0.02	2.47	0.35 to 17.5	0.36

### Secondary outcomes: Course of disease and antibiotic use

Number of days of hospitalization among the admitted patients was mean 7.3 days (median 6, range 1–35) before the intervention and mean 7.1 days (median 5, range 1–30) after the intervention, (P = 0.9). Patients provided intravenous antibiotics were treated mean 7.3 days (median 6.0, range 1–29) before and mean 8.2 days (median 7.0, range 1–36) after the intervention (P = 0.30). Patients provided intravenous fluids were treated mean 3.8 days (median 3.5, range 1–11) before and mean 4.4 days (median 3.0, range 1–30) after the intervention (P = 0.43).

### Nursing home versus hospital treatment

The patients were treated with intravenous antibiotics mean 7.5 days (median 6, range 1–25) in the nursing homes, mean 8.4 days (median 6, range 1–36) in the hospital (P = 0.21). Among patients receiving intravenous antibiotics in the nursing homes, 50 (45% (36 to 55%)) died within 30 days, compared to 30 (36%, 25 to 46%) treated in the hospital (P = 0.17).

Patients provided intravenous fluids were treated mean 4.7 days (median 4, range 1–30) in the nursing homes, mean 2.2 days (median 2, range 1–5) in the hospital (P = 0.01). Among patients receiving intravenous fluids locally, 21 (19%, 95% CI 11 to 26%) died within 30 days, compared to 2 (8%, 95% CI 0 to 20%) in the hospital group (P = 0.22).

Multilevel logistic regression analysis revealed that treatment with intravenous antibiotics rather than fluids, reduced level of consciousness, elevated CRP-value and age <70 were associated with 30-day mortality (Table 5). Treatment located in nursing home or in hospital was not associated with increased mortality. In identical analysis on the subgroup receiving intravenous antibiotics, reduced level of consciousness and age <70 was associated and elevated CRP-value was insignificantly associated with 30-day mortality. In the analysis on the subgroup receiving intravenous fluids, no factors was significantly associated with 30-day mortality.

**Table 5:** Associations of demographic and clinical variables with 30-day mortality in patients provided intravenous treatment in nursing homes and hospital. Multilevel logistic regression model with nursing home as cluster (random intercept).

	Bivariate analysis			Multivariate analysis (n = 249)		
	OR	(95% CI)	P-value	OR	(95% CI)	P-value
**Intervention**	1.49	0.88 to 2.51	0.14	1.22	0.50 to 3.05	0.66
**Nursing home treatment**	1.12	0.68 to 1.84	0.67	1.43	0.64 to 3.23	0.39
**Intravenous antibiotics**	3.54	2.05 to 6.11	<0.01	2.79	1.23 to 6.30	0.01
**Female**	1.10	0.69 to 1.76	0.69	1.03	0.56 to 1.90	0.92
**Age**						
<70	Reference			Reference		
70 to 79	0.31	0.13 to 0.70	<0.01	0.17	0.57 to 0.52	<0.01
80 to 89	0.42	0.20 to 0.88	0.02	0.37	0.13 to 1.04	0.06
>90	0.37	0.16 to 0.88	0.02	0.37	0.12 to 1.14	0.08
**Congestive heart failure**	0.22	0.72 to 2.09	0.46	1.41	0.72 to 2.77	0.32
**Reduced consciousness**	2.14	1.31 to 3.47	<0.01	2.61	1.41 to 4.83	<0.01
**Systolic blood pressure at onset (tertiles)**						
<115 mmHg	Reference			Reference		
115 to 138 mmHg	0.87	0.50 to 1.53	0.63	1.28	0.61 to 2.68	0.52
>138 mmHg	0.71	0.39 to 1.27	0.25	0.93	0.43 to 2.00	0.86
**CRP at onset (tertiles)**						
<65	Reference			Reference		
65 to 156	1.28	0.67 to 2.44	0.46	0.86	0.39 to 1.93	0.72
>156	2.98	1.59 to 5.57	<0.01	1.84	0.82 to 4.15	0.14
**Number of nursing homes in intervention (time factor)**						
1 to 5	Reference			Reference		
6 to 10	0.53	0.22 to 1.25	0.15	0.54	0.17 to 1.68	0.29
11 to 15	1.41	0.65 to 3.06	0.38	1.86	0.64 to 5.42	0.26
16 to 20	0.70	0.33 to 1.47	0.34	0.67	0.24 to 1.89	0.45
21 to 25	0.76	0.31 to 1.87	0.56	0.95	0.25 to 3.57	0.94
26 to 30	0.73	0.35 to 1.52	0.40	0.71	0.22 to 2.32	0.57

### Antibiotic choice

The choice of intravenous antibiotics differed in the nursing homes compared to the hospital (Table 6). For pneumonia, 47 (62%, 51 to 73%) of 76 nursing home patients were given cephalosporins alone or in combinations, 18 (38%, 24 to 53%) of 47 patients treated in the hospital (P = 0.01). For urine tract infections, 12 (92%, 76 to 100%) of 13 nursing home patients were given cephalosporins, 11 (85%, 34 to 64%) of 13 patients treated in the hospital (P = 0.54). The antibiotic choice was also broad spectrum among the 52 patients who were provided intravenous fluids and oral antibiotics, but numbers were too small to compare choices in nursing homes versus hospital. Phenoxymetylpenicillin was provided to only 6 (12%, 3 to 21%) of 52 patients, all with a respiratory tract infection (Table 6).

**Table 6:** Choice of antibiotics in nursing homes versus hospital to patients provided intravenous and oral antibiotics, by diagnosis.

	**Iv antibiotics in nursing homes (n = 110)**				**Iv treatment in hospital (n = 84)**				**Total**
	**RTI**	**RTI + UTI**	**UTI**	Other*	**RTI**	**RTI+UTI**	**UTI**	Other*	
Benzylpenicillin	26 (34)	1 (10)	0	2 (18)	23 (49)	4 (19)	0	0	56 (29)
Broad spectrum penicillin	1 (1)	1 (10)	0	0	3 (6)	1 (5)	2 (15)	0	8 (4)
Cefalosporins	43 (57)	8 (80)	12 (92)	7 (64)	17 (36)	12 (57)	10 (77)	1 (33)	110 (57)
Other iv antibiotics**	2 (3)	0	1 (8)	0	0	1 (5)	0	0	4 (2)
Combinations***	4 (5)	0	0	2 (18)	4 (9)	3 (14)	1 (8)	2 (67)	16 (8)
Total	76 (100)	10 (100)	13 (100)	11 (100)	47 (100)	21 (100)	13 (100)	3 (100)	194 (100)
	**Oral antibiotics in nursing homes (n = 44)**				**Oral antibiotics in hospital (n = 8)**				**Total**
	**RTI**	**RTI + UTI**	**UTI**	Other*	**RTI**	**RTI+UTI**	**UTI**	Other*	
Phenoxymetylpenicillin	6 (30)	0	0	0	0	0	0	0	6 (12)
Broad spectrum penicillin	10 (50)	1 (33)	8 (50)	2 (40)	1 (50)	0	2 (40)	0	24 (46)
Ciprofloxacin	1 (5)	0	4 (25)	0	0	1 (100)	3 (60)	0	9 (17)
Doksycyclin	3 (15)	0	0	0	0	0	0	0	3 (6)
Nitrofuradantin	0	0	2 (13)	0	0	0	0	0	2 (4)
Trimetoprim +/- sulfa	0	1 (33)	1 (6)	0	1 (50)	0	0	0	3 (6)
Other oral antibiotics****	0	1 (33)	1 (6)	3 (60)	0	0	0	0	5 (10)
Total	20 (100)	3 (100)	16 (100)	5 (100)	2 (100)	1 (100)	5 (100)	0	52 (100)

*Other: skin, gastrointestinal and unspecified infections;

**Other iv antibiotics: ciprofloxacin (n = 1), meropenem (n = 3);

***Combinations: cefotaksim + benzylpenicillin/metronidazol/meropenem/klindamycin (n = 9), gentamicin + benzylpenicillin/ampicillin (n = 5), klindamycin + benzylpenicillin (n = 2);

****Other oral antibiotics: cefotaksim (n = 1), erythromycin (n = 1), metronidazol (n = 2), vancomycin (n = 1).

### Implementation of intravenous treatment in the nursing homes

Over 90% of the health personnel (nurses and nursing assistants) in the 30 nursing homes received the intervention. Feedback during the training, follow-up meetings and evaluations was positive. Among advantages described, were that the patients were treated in surroundings and by personnel familiar to them—by personnel knowing them well; and the general phrase: “That hospitalization was avoided”. Among disadvantages described were practical difficulties with placing and keeping the PVC and confrontation with ethical dilemmas with end-of-life treatment. As a solution to the former, the ambulance service offered to assist in the practical problems with the PVC when necessary. All nursing homes were actively planning to continue providing intravenous treatment in the future.

## Discussion

The principal finding of this trial were that a structured training program in administrating intravenous fluids and antibiotics was highly effective in reducing the number of hospital admissions for dehydration and infections among nursing home residents. Hospitalization of the acute ill and frail elderly patient in many cases lead to a worsening of functional abilities, even though the specific condition for which the patient is transferred may improve [20, 21]. The research literature on hospitalization from nursing homes is extensive, but it is difficult to generalize on the extent of avoidable complications of hospital transfers such as delirium and pressure ulcers [11]. However, given that the patient can receive the same treatment and care in the nursing home, to avoid the burden and complications of relocation is obviously beneficial to the patients.

The total number of patients provided intravenous treatment fell during the project period: the proportion of patients treated in hospital reduced during the intervention while the number treated in nursing homes was stable. Of the factors contributing to the overall reduction is an unplanned effect of the intervention: The nursing home staff described an increased awareness and increased use of advanced care planning, as well as more informal general and case specific discussions of ethical aspects and actual need of intravenous treatment and of hospitalization; leading to a more prudent consideration of curative or supportive treatment of the patients. The proportion of patients provided intravenous fluids was higher in the nursing homes than in the hospital, indicating that the need was higher and threshold lower for providing intravenous fluids than for parenteral antibiotic treatment locally. Length of intravenous treatment, days of hospitalization among the admitted patients and 30-day mortality before and after the intervention, was similar. 30-day mortality was not associated with location of treatment, but the study was underpowered to conclude on mortality among patients provided intravenous treatment in nursing homes versus hospital. Factors associated with increased 30-day mortality were treatment with intravenous antibiotics rather than fluids, CRP > 165 and reduced consciousness, all being factors serving as a proxy for severity of disease; also found elsewhere [14, 22]; and age <70. In the nursing home population, biological age is not a predictor of prognosis, but a higher mortality in the youngest residents is likely because these are often patients with cancer in palliative units or patients with severe and invalidating diseases.

The Norwegian guidelines for empirical treatment of pneumonia state that benzylpenicillin is the first-line antibiotics for empirical treatment in both nursing homes and hospital [17, 18]. Two thirds of the parenteral antibiotics used in this study was broad-spectrum, and the majority of patients in nursing homes and in hospital were given 3^rd^ generation cephalosporins. A safe and effective strategy for antibiotic use involves prescribing antibiotics only when it is needed and selecting appropriate and effective medicines with the narrowest spectrum of antimicrobial activity. The spread of MRSA in nursing homes has been reported in a number of countries including Norway [23]; and infections caused by ESBL-producing bacteria is a rising problem [24–26]. The emergence of resistant bacteria argues for careful prescription of antibiotics as well as restricting transfer of patients between nursing homes and hospital when it is not necessary.

The principal strength of this study is its size and design: the stepped wedge cluster randomized trial is a pragmatic study design, which can enable research on planned service delivery interventions without compromising with the concerns of the stakeholders, in this case, the rollout of an educational program planned by the regional hospital. The design allowed for implementation approximately as planned as well as a randomized evidence of effectiveness. The intervention rolled out as planned without unexpected challenges and the education was provided to almost all personnel in the nursing homes. We included all cases of intravenous treatment in which hospital admissions could be avoided, both patients with serious infections and cases of dehydration; and the follow up for all the patients for 30 days made it possible to assess a prognosis. The study nursing homes were the vast majority of public and private nursing homes in one county, and probably without relevant differences from Norwegian nursing homes in general. The intervention itself can be repeated without large investments or resources; it did not require more equipment than a training arm and intravenous therapy supplies and was carried out by two nurses.

The study’s main limitation was the difficulties collecting data. Not all patients treated locally were included in the study and the data collection forms were incomplete for a number of patients. We have no reason to believe that patients not included in the study differed from patients included, or that the main results could have been altered, but it may have lead to an underestimate of the need for intravenous treatment among nursing home patients. We have no reason to believe that a systematic change in under-reporting over time has lead to a fictive time trend of reduced intravenous treatments throughout the study period. A second limitation was that although we through the inclusion criteria aimed to ensure comparability between the patients treated in nursing homes and the patients admitted to hospital, the two groups are not identical. We assume that in the study as well as in clinical practice, there is a trend towards more seriously ill patients being hospitalized, shown by the higher proportion of patients with congestive heart failure, high blood pressure and high CRP in the hospital group. However, among patients given intravenous treatment locally, there will be some that are provided intravenous treatment as palliative care in a terminal phase who would not been hospitalized for the same treatment. How this affects the outcomes of the study is difficult to assess, but may have contributed to a higher mortality in the nursing home group. A further limitation is that the two pilot nursing homes had no observational time, and due to low turnover of intravenous treatment, eight additional nursing homes had data for one level only, resulting in a certain loss of power. Last, the original power calculation was for a standard randomized controlled trial, allowance for the number of steps and allowance for any repeated measures on individuals was not included in this sample size estimate.

This study is the first to evaluate the effect of a training program in intravenous treatment in nursing homes using a stepped-wedge design. The composition of the nursing home population, and views and traditions on optimal care, treatment strategies and treatment intensity among nursing home residents, vary across countries [6], making comparisons with other studies, recommendations for further research as well as policy recommendations challenging. Different aspects of the topic “hospitalization from nursing homes” have been elucidated in the research literature the last decades [11]. We have only identified two studies using the stepped wedge approach in the nursing home settings, none on intravenous treatment or on reducing hospitalization [27, 28]. Several interventions to structure or standardize clinical practice have been evaluated [12]. In Canada, Loeb et al. found that a clinical pathway for on-site treatment of pneumonia and other lower respiratory tract infections in nursing homes resulted in comparable clinical outcomes and reduced hospitalizations and health care costs [9]. A multifaceted intervention study to implement guidelines in the USA did not affect hospitalization rates for nursing home-acquired pneumonia [10]. A previous USA-based study found that an education intervention directed at guidelines on antibiotic treatment in nursing homes was feasible and increased adherence to treatment guidelines, but had no effect on hospitalization or 30-day mortality [7]. The effect on hospitalization of the training program implemented among nursing home personnel in this study may be partly explained by elevated medical competence in the nursing homes: both an increased theoretical knowledge of both prevention and treatment of infections and dehydration, and competence in an essential practical treatment procedure; partly by the described increased awareness regarding advance care planning.

Due to demographic changes and an intensified effort in community care of the elderly [29], residents in European nursing homes have over the past decades become increasingly frail, often with multiple active diagnoses [30], and the situation is similar in Norway. In addition, in 2012, a political reform was introduced in Norway, aiming at treatment on lowest effective level of care, including an increased focus on the interaction between hospitals and nursing homes. Following, the burden of disease in the nursing homes have increased, making it necessary for the nursing homes to increase their medical competence [31]. However, the need for intravenous treatment among nursing home patients is limited. Pre-intervention, many of the long term facility leaders and -workers were skeptic to the intervention, arguing that it would be too resource demanding. It became apparent through the project period that the need for intravenous treatment was low in the majority of nursing homes; exceptions were short term, palliative and intensive care units. After intervention, 22 of 30 nursing homes had fewer than 10 patients per 100 beds per year receiving intravenous treatment, and all nursing home leaders confirmed that local intravenous treatment in most facilities was feasible without large reallocations of existing resources. Further, the hospital reports obvious benefits of the intervention: less pressure from hospitalizations and more effective discharges as the nursing homes can continue initiated intravenous treatment to stabilized patients.

Health care policies around the globe are seeking ways to increase efficacy and reduce strain on specialist health care, and reducing emergency admissions is often accentuated as the key to achieve this [12]. Increased evidence on interventions reducing hospital admissions from nursing homes have been explicitly requested [12]. This study fills some of the evidence-policy-gap and can contribute to inform current policies and future reforms. Our study demonstrated that it is feasible to do a pedagogic intervention by use of a stepped wedge design. The significant effect of the structured training program in intravenous treatment in nursing homes makes the intervention almost directly recommendable for nursing homes in Norway. We clearly recommend evaluating this intervention adapted to nursing homes in other settings and other countries, as one strategy to reduce hospital admissions. Future research should also incorporate barriers and facilitators for local management of nursing home patients both on individual and structural level.

## Supporting information

S1 Fig Number of patients receiving intravenous antibiotics and fluids in nursing home versus hospital in the study period, per 3 months. Pilot sites not included.
(JPG)

S1 Table Associations of demographic and clinical variables with intravenous treatment in the nursing home. Multilevel logistic regression model with nursing home as cluster (random intercept). Pilot sites not included.
(DOC)

S1 Appendix Project protocol. (DOCX)

S2 Appendix Research form. Basic form for all patients.
(DOC)

S3 Appendix Research form. Form for patients treated with iv antibiotics in hospital.
(DOC)

S4 Appendix Research form. Form for patients treated with iv antibiotics in nursing homes.
(DOC)

S5 Appendix Research form. Form for patients treated wit iv fluids in hospital.
(DOC)

S6 Appendix Research form. Form for patients treated wit iv fluids in nursing homes.
(DOC)

S7 Appendix Consent form. (DOC)

S8 Appendix TIDieR-checklist. (DOCX)
