# Supplementary figures and images for: A structured training program for health workers in intravenous treatment with fluids and antibiotics in nursing homes: A modified stepped-wedge cluster-randomised trial to reduce hospital admissions
Source: PLoS One. 2017 Sep 7;12(9):e0182619. doi: 10.1371/journal.pone.0182619 (PMC5589147; doi:10.1371/journal.pone.0182619)

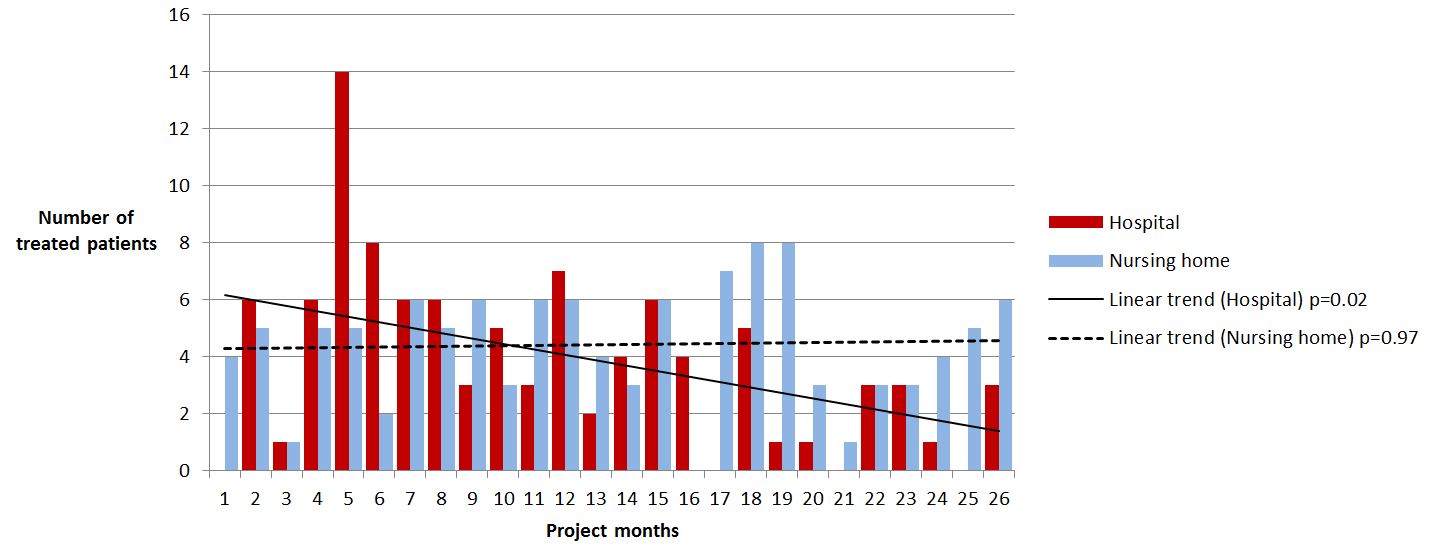

Supplement: S1 Fig — Pilot sites not included. (JPG) [file pone.0182619.s001.JPG]
