# Supplementary material for: A structured training program for health workers in intravenous treatment with fluids and antibiotics in nursing homes: A modified stepped-wedge cluster-randomised trial to reduce hospital admissions
Source: PLoS One. 2017 Sep 7;12(9):e0182619. doi: 10.1371/journal.pone.0182619 (PMC5589147; doi:10.1371/journal.pone.0182619)
Supplement: S1 Table — Multilevel logistic regression model with nursing home as cluster (random intercept). Pilot sites not included. (DOC) [file pone.0182619.s002.doc]

**Table 4** Associations of demographic and clinical variables with intravenous treatment in the nursing home. Multilevel logistic regression model with nursing home as cluster (random intercept). Analysis without pilots included (n=213).

| **Factors** | **Bivariate analysis** | | | **Multivariate analysis (N=154)** | | |
| --- | --- | --- | --- | --- | --- | --- |
|  | **OR** | **(95 % CI)** | **P-value** | **OR** | **(95 % CI)** | **P-value** |
| **Intervention** | 4.84 | 2.40 to 9.76 | <0.01 | 5.58 | 1.34 to 23.12 | 0.018 |
| **Intravenous antibiotics** | 0.16 | 0.08 to 0.35 | <0.01 | 0.68 | 0.21 to 2.18 | 0.52 |
| **Gender** | 0.65 | 0.34 to 1.24 | 0.19 | 0.82 | 0.28 to 2.39 | 0.71 |
| **Reduced consciousness** | 3.35 | 1.69 to 6.64 | <0.01 | 2.01 | 0.71 to 5.71 | 0.19 |
| **Systolic blood pressure at onset (tertiles)**  <115 mmHg  115 to 138 mmHg  >138 mmHg | Reference  0.59  0.26 | 0.26 to 1.34  0.12 to 0.57 | 0.21  <0.01. | Reference  0.96  0.25 | 0.26 to 3.60  0.08 to 0.85 | 0.96  0.03 |
| **CRP at onset (tertiles)**  <65  65 to 156  >156 | Reference  0.67  0.28 | 0.30 to 1.49  0.12 to 0.70 | 0.33  <0.01 | Reference  0.33  0.17 | 0.15 to 1.82  0.06 to 1.00 | 0.31  0.05 |
| **Congestive heart failure** | 0.35 | 0.15 to 0.81 | <0.02 | 0.29 | 0.09 to 0.96 | 0.04 |
| **Number of nursing homes in intervention (time factor)**  1 to 5  6 to 10  11 to 15  16 to 20  21 to 25  26 to 30 | Reference  0.41  1.13  0.71  3.57  3.04 | 0.13 to 1.25  0.39 to 3.26  0.21 to 2.43  0.84 to 15.19  0.94 to 9.83 | 0.12  0.82  0.59  0.09  0.06 | Reference  0.28  1.15  0.74  3.63  1.80 | 0.03 to 2.71  0.13 to 10.05  0.07 to 8.36  0.22 to 60.03  0.14 to 22.69 | 0.27  0.90  0.81  0.37  0.65 |
