# Supplementary material for: A structured training program for health workers in intravenous treatment with fluids and antibiotics in nursing homes: A modified stepped-wedge cluster-randomised trial to reduce hospital admissions
Source: PLoS One. 2017 Sep 7;12(9):e0182619. doi: 10.1371/journal.pone.0182619 (PMC5589147; doi:10.1371/journal.pone.0182619)
Supplement: S1 Appendix — (DOCX) [file pone.0182619.s003.docx]

Protocol 3IV-study

Can a structured training program in intravenous treatment of infections in nursing homes lead to a better patient trajectory and reduced hospital admittance?

Importance

Det er over 900 sykehjem og 40 000 sykehjemsbeboere i Norge.There are over 900 nursing homes and 40 000 nursing home residents in Norway. Gjennomsnittsalderen på beboerne er 84 år, og 73% er kvinner. The average age of a resident is 84, and 73 % are women. Pasientene i sykehjem har 5–7 diagnoser hver, og sykdomsbildet hos eldre er komplisert (Helling 2008). Patients in nursing homes each have 5.7 diagnoses, and the diseases in the elderly are complex (Helling 2008). Til enhver tid blir 5-8% av beboerne behandlet med systemiske antibiotika (Andersen 2002, Tobiassen 2002), og over 50% vil i løpet av et år få antibiotika for en infeksjon (Tobiassen 2002). At any time 5.8 % of the residents are treated with systemic antibiotics (Andersen 2002, Tobiassen 2002), and over 50 % will in the course of a year receive antibiotics for an infection (Tobiassen 2002). Omfanget gjør optimalisering av behandling med antibiotika blant sykehjemspasienter til en viktig problemstilling i primærhelsetjenesten både medisinskfaglig og ressursmessig. These proportions make an optimization of the treatment with antibiotics among nursing home patients an important subject matter within primary health care, both in regards to medicine as well as resources.

Quality assurance and patient safety aspects

Dette prosjektet tar utgangspunkt i at mange sykehjemspasienter må legges inn på sykehus for å motta behandling med intravenøs væske og/eller antibiotika. Kunne flere av disse pasientene vært behandlet på sykehjem med like gode resultater?This project is based on the fact that many nursing home patients must be hospitalized in order to receive treatment with intravenous fluids and/or antibiotics. Hospitalization may be a burden for many nursing home patients. The 3IV study will provide a training program in intravenous treatment to nursing homes in Vestfold county and evaluate the intervention by following patients who are treated in nursing homes and in hospital. In this way we will be able to answer a number of questions about intravenous treatment in nursing homes. Can more of these patients have been treated in a nursing home with equally good results? Vil det redusere belastningen for pasientene og ressursbruken for samfunnet? Will this reduce the burden for the patients and the use of resource for the society? Hva skal til av ressurser og kompetanse ved sykehjemmene for å få til dette og eventuelt hva slags samhandling vil eventuelt være nødvendig med nærmeste sykehus? Which resources and expertise are needed at the nursing home in order to execute this, and what kind of interaction with the nearest hospital would then be necessary? In the 3IV intervention we will also focus on ethical dilemmas when nursing home patients become seriously ill.

BakgrunnBackground

Det er en høy prevalens av bakterielle infeksjoner i sykehjemsbefolkningen, hovedsakelig i luft- og urinveier (Tobiassen 2002, Loeb 2005, Stone 2001).There is a high prevalence of bacterial infections in the nursing home population, mainly linked to respiratory and urinary disorders (Tobiassen 2002, Loeb 2005, Stone 2001). Eldre mennesker som er syke blir ofte dehydrerte på grunn av svekket allmenntilstand og vansker med å ta til seg tilstrekkelig med væske. Elderly who are sick often get dehydrated due to a poor general condition and difficulties with consuming enough liquid. Infeksjoner kan utvikle seg raskt slik at det blir behov for raskt innsettende behandlingseffekt av antibiotika – som derfor mest optimalt kan tilføres intravenøst. Selve *administrasjonsmåten* er ofte eneste årsak til at mange sykehjemspasienter transporteres til sykehus for å motta behandling med væske eller antibiotika som i prinsippet kunne vært gitt i sykehjem. Infections can progress rapidly, something which makes a swiftly responding treatment with antibiotics necessary. For the most optimal effect this should be given intravenously. The way this procedure is executed, is very often the single reason why many nursing home patients are transported to a hospital to receive treatment with fluids or antibiotics, something which could easily have been done in the nursing home.

Sykehjemmene er i dag pleieinstitusjoner underlagt omsorgstjenesten i kommunene, og tradisjonelt har pleie og omsorg vært prioritert høyere enn det medisinskfaglige tilbudet til beboerne (Helling 2007).The current nursing homes are institutions under subordination of the care service of the municipalities where nursing and care giving as a tradition always have been a higher priority rather than medical service to the residents (Helling 2007). Unødvendige innleggelser i sykehus kan ha med mange faktorer å gjøre, deriblant organiseringen av legetilbudet og med finansieringssystemet for helsetjenester til sykehjemspasienter (Ranhoff 2005). Unnecessary hospital admissions could be due to many factors, including the organization of the GP service and the financing of health services to nursing home patients (Ranhoff 2005). En allmennlege med tilsynslegefunksjon er vanligvis tilgjengelig bare en eller og dager i uken, og akutte tilstander blir tatt hånd om av legevaktsleger. A general practitioner with a supervising function is usually only available once or twice a week and emergency situations are taken care of by ER doctors. Medisinsk virksomhet er, i motsetning til på sykehus, ikke underlagt noen form for refusjonsrett, takster eller DRG-poeng, og alle utgifter til behandling belastes kommunen. I dag har vi et system som gjør at det som er billig, godt og riktig medisinsk sett er utgiftsgenererende for kommunene (Høie 2009). Medical work is in contrast to hospital work not subject to any reimbursement, tariffs or DRG-points, and treatment costs are charged the municipality. Today we have a system which makes everything that is inexpensive, good and proper medicine into expenditure for the municipalities (Høie 2009).

Mange pasienter kunne vært behandlet like godt på sykehjemmet uten unødvendig flytting, med lavere bruk av ressurser og med like god eller bedre prognose (Boockvar 2005, van der Steen 2004, Fried 1997, Fried 1995).Many patients could have been treated just as well in the nursing home without any unnecessary transportation, less use of resources and with the same or better prognosis (Boockvar 2005, van der Steen 2004, Fried 1997, Fried 1995). Sykehus har fokus på diagnostikk og aktiv behandling, mens pleiekomponenten kompromitteres. Hospitals tend to focus on diagnostics and active treatment while the care aspect is compromised. Dette kan resultere i mangelfull ernæring, liggesår, permanent blærekateter og andre komplikasjoner - som alle bidrar til å forlenge den aktuelle sykdommen. This can lead to malnutrition, decubitus, permanent bladder catheter and other complications - all of which contributes to an extension of the existing disease. Et vesentlig problem ved innleggelse fra sykehjem til sykehus er faren for forvirringstilstander hos pasientene. A significant problem upon admission from nursing homes into hospitals is the danger of a state of confusion in the patients. Fra studier i USA er det vist at varighet av delir er en viktig prediktor for mortalitet det kommende året (Kielv 2009). Studies from the US show that the duration of delirium is an important predictor for mortality in the upcoming year (Kielv 2009). Table 1 sums up potential advantages and disadvantages of providing intravenous treatment in the nursing homes versus in hospital.

Tabell 1: Fordeler og ulemper ved intravenøs behandling i sykehjem og på sykehus **Table 1** Potenital advantages and disadvantages of intravenous treatment in nursing homes and in hospital

| **Intravenøs behandling med væske og/eller antibiotika på sykehjem** **Intravenous treatment with fluids and / or antibiotics in nursing homes** |
| --- |
| *Fordeler Benefits*  Pasienten får være i sine vante omgivelserThe patient will be in familiar surroundings  Bedre pleie og omsorgBetter continuity and holistic approach when the health personnel knows the patients  Better nursing and care giving?  Ressursbesparende totalt settAn overall saving of resources  Less pressure on hospital admissions, fewer hallway patients  Improved preparedness for the ambulance service |
| *Ulemper Disadvantages*  Ressurskrevende på sykehjemsnivå An increased need for resources in nursing homes including equipment and manpower  A need for improved medical competence and skills |
| **Intravenøs behandling med væske og/eller antibiotika på sykehus** **Intravenous treatment with fluids and/or antibiotics in hospitals** |
| *Fordeler Benefits*  Mest mulig presis diagnostikk og oppfølging av den aktuelle tilstanden: blodprøver, røntgenOptimal diagnostic and monitoring of the condition: blood tests, x-ray  Mapping of co-morbidity and complicating factors such as reduced lung- and renal function |
| Ulemper *Disadvantages*  Kan utløse forvirring og uro hos pasienten (hva vet vi om dette?)CCan trigger confusion and anxiety in the patient  Complications related to inadequate nursing and care  Possible nursing home infections and the danger of a spreading of multi-resistant bacterialbakteriestammer til sykehjem strains to the nursing home Komplikasjoner knyttet til mangelfull pleie og omsorg |

Det er nødvendig med en opprustning av sykehjemmene for å kunne gi intravenøs behandling lokalt.AAAA strengthening of the nursing homes is required in order to provide intravenous treatment locally. Kompetanse hos sykepleiere og involverte allmennleger og utstyr og apparatur for supplerende undersøkelser (CRP, O _2_ -metning, urin stix og –dyrkning etc.) er en forutsetning for god diagnostikk og behandling. Enkelte sykehjem tilbyr allerede intravenøs behandling til sine beboere. Etter det økte fokus på samhandling har det blitt gjennomført samarbeidsprosjekter mellom sykehus og sykehjem for å tilby intravenøs behandling til sykehjemsbeboere lokalt. Competent nurses and GPs and equipment and apparatus for supplementary examinations (CRP, O_2_ –saturation, urine sticks and cultivation, etc.) are essential for proper diagnostics and treatment. Some nursing homes already offer intravenous treatment to their residents. After an increased focus on interaction, collaborative projects between hospitals and nursing homes with the purpose of providing intravenous therapy locally to nursing home residents have been carried out. Ved hjelp av relativt små opplæringstiltak har dette vist seg å være gjennomførbart blant annet i kommuner i Østfold og Telemark. Det har ikke vært gjennomført systematiske evalueringer av intravenøs behandling ved sykehjem i Norge og i begrenset grad i utlandet. Through quite simple teaching methods this has proven feasible in among others municipalities in Østfold and Telemark. A methodical evaluation of intravenous treatments has never been executed in Norway before and to a very limited extent in the rest of the world. Vi har funnet en review fra USA som beskriver to prospektive observasjonsstudier og 2 retrospektive case-control studier på behandling av sykehjemsbeboere med lungebetennelse. We have found one review from the United States which describes two prospective observational studies and two retrospective case-control studies on the treatment of nursing home residents with pneumonia. Den oppsummerer at sykehusinnleggelse for denne gruppen pasienter ikke er nødvendig og fører til økte kostnader, morbiditet og dødelighet – men at mer forskning er nødvendig (Dosa 2005). The review concludes that hospitalization for this group of patients is not necessary and leads to increased costs, morbidity and mortality, but that further research are necessary. (Dosa 2005).

**Patient autonomy in medical decisions**

According to Norwegian legislation, patients’ informed consent is a prerequisite to provide medical treatment - except emergency care. If the patient is incapable of giving consent, treatment can be provided if it is considered to be to the best for the patient and if it is probable that the patient would have consented to the treatment. Relatives should be included in the decision-making process mainly because of their assumed knowledge of the patient’s will.

In the 3IV intervention we have included two simple measures focusing on ethical challenges. The main objectives are 1) to improve the management of ethical questions early in the process and during acute illness among elderly people in nursing homes in Vestfold and 2) increased knowledge about patient autonomy with regards to decisions about life-prolonging treatment in nursing homes.

We will focus on assessment and documentation of decision making capacity; conversations about treatment with patients and relatives in advance and during acute illness, and the decision-making process concerning life-prolonging treatment at the end of life

Prosjektets hovedmål og delmålPrimary and secondary objectives for the 3 IV-project

Prosjektets hovedmål er å evaluere om sykehjemsbeboere som trenger intravenøs væske og intravenøs antibiotika kan behandles like godt eller bedre på sykehjemmet som i sykehus.The *primary objective* for the project is to evaluate whether nursing home residents who require intravenous fluids and intravenous antibiotics, can be treated just as well or even better in the nursing home as in the hospital.

Prosjektets delmål er som følger: The project's *secondary objectives* are as follows:

1. Kan et strukturert opplæringsprogram i intravenøs væske- og antibiotikabehandling føre til redusert antall innleggelser og liggedøgn i sykehus blant sykehjemsbeboere?Can a structured training program in administration of intravenous fluids and antibiotics lead to a reduced number of admissions and days in hospitals among nursing home residents?
2. CKan behandling med intravenøs væske eller antibiotika på sykehjemmet gi et like godt eller bedre pasientforløp sammenlignet med pasienter som blir innlagt på sykehus for denne behandlingen?an treatment with intravenous fluids or antibiotics in nursing homes provide an equally good or better patient trajectory compared to patients hospitalized for the same treatment?

Vi vil spesielt se på a) varighet av sykdomsforløp We will take a closer look at a) Duration of symptoms b) direkte og indirekte komplikasjoner (forvirring og delir, b) Direct and indirect complications (confusion and delirium, liggesår, trombo-embolisk sykdom, falltendens etc.)decubitus, trombo- embolic disease, fall frequency, etc.) c) mortalitetc) Mortality 3. Kan behandlingskostnadene for helsevesenet reduseres når pasienter behandles med intravenøs væske og/eller antibiotika på sykehjemmet framfor å bli innlagt på sykehjem?Can treatment costs for the public health service be reduced when patients are treated with intravenous fluids and /or antibiotics in the nursing home instead of being admitted to the hospital?

4. 4. Can treatment for dehydration and infections in the nursing home result in a similar or better sTilfredshet hos pasienter, pårørende og hos sykehjemspersonelletatisfaction among patients, next of kin and in nursing personnel?

Secondary objectives concerning *patient autonomy* and *ethical issues*

1. Increased knowledge of patient autonomy in decisions concerning life prolonging treatment in nursing homes
2. To contribute to a better management of ethical issues in advance and in acute illness among nursing home patients

We will focus on the following

- 1. For how many patients has the competence of consent been assessed?
  2. How many patients have participated in a preparatory conversation?
  3. How are decisions concerning life prolonging treatment handled in acute situations?

MetodeMethod

Studien er designet som en cluster-randomisert studie hvor randomisering skjer på sykehjemsnivå. Den vil følge et 'stepped wedge-design' (ref) hvor hvert sykehjem er i kontrollgruppen før inklusjon og i intervensjonsgruppen etter intervensjon.The study is designed as a cluster-randomized study in which the randomization takes place on a nursing home level. It will follow a ”stepped wedge design” (MRC 2006), where each nursing home is in the control group before inclusion and in the intervention group after intervention. Rekkefølgen for inklusjon av det enkelte sykehjem blir randomisert. The order for the inclusion of the individual nursing homes will be randomized. Intervensjonen er et strukturert opplæringsprogram i intravenøs behandling av dehydrering og infeksjoner i sykehjem. The intervention is a structured educational program in intravenous treatment of dehydration and infections in nursing homes.

**The training program in intravenous treatment in nursing homes**

Intervensjonen skal gjennomføres ved de 28 kommunale sykehjemmene i Vestfold fylke.The intervention will be implemented in the 34 municipal nursing homes in the county of Vestfold. Oppstart av infeksjonsregistreringer vil starte på samme tidspunkt for alle sykehjem og er planlagt til oktober 2009. The starting point for the registration of infections will begin on the same date for all the nursing homes and is scheduled for October 2009. Inklusjon av sykehjem vil pågå i 18 måneder, som innebærer at ett til to sykehjem inkluderes per måned. The inclusion of nursing homes will continue for 18 months, which means that one or two nursing homes will be included each month. Vi vil registrere alle infeksjoner ved sykehjemmene i inklusjonsperioden. We will record all infections in the nursing home during the inclusion period. Ved sykehjem som har gjennomgått opplæringsprogrammet, vil pasienter som trenger væske eller antibiotika intravenøst bli behandlet på sykehjemmet – forutsatt tilstrekkelig kapasitet og kompetanse der og da. In nursing homes which have completed the training program, patients who are in need of intravenous fluids or antibiotics will be treated at the nursing home, provided sufficient capacity and expertise are in place. Kriterier på pasientnivå for behandling i sykehjem er beskrevet i Tabell 2. The criteria for patient-level treatment in nursing homes are described in Table 2. Ved sykehjem som ikke har gjennomgått opplæringsprogrammet, vil pasienter som trenger væske og antibiotika intravenøst bli innlagt på sykehus. In nursing homes which have not completed the training program, patients in need of intravenous fluids and antibiotics will be hospitalized. Hos alle pasienter som blir behandlet med intravenøs væske og/eller antibiotika i sykehjem eller på sykehus, vil det bli anmodet om samtykke til å hente detaljert informasjon om forløpet. All patients treated with intravenous fluids and / or antibiotics in nursing homes or hospitals, will be asked for a consent to collect detailed information about the progress. Pasienter med sepsisutvikling eller komorbiditet som krever sykehusinnleggelse uansett, ekskluderes fra analysene. Patients who develop sepsis or have co-morbidity which requires hospitalization, will be excluded from the analysis.

Tabell 2: Kriterier på pasientnivå for behandling med intravenøs antibiotika på sykehjem

**Table 2** Criteria for patient-level treatment with intravenous antibiotics in nursing homes

| **Behandling på sykehjemmet** **Treatment in nursing home** |
| --- |
| DehydrationLungebetennelse, urinveisinfeksjon eller hudinfeksjon diagnostisert på sykehjemmet, pneumonia, urinary tract infection or skin infection diagnosed in the nursing home ved hjelp av på symptomer, klinisk undersøkelse og enkle tester (CRP, oksygenmetning)using symptoms, clinical examination and simple tests (CRP, oxygen saturation)  Behov for intravenøs behandling (per oral behandling ikke tilstrekkelig) Need for intravenous treatment (per oral treatment is not sufficient)  Necessary competence and capacity in the nursing home |
| **Innleggelse på sykehus** **Hospitalization** |
| KomorbiditetSSerious infection (sepsis) or need for more advanced diagnostics  Other diseases/co-morbidity that in itself requires hospitalization Alvorlighetsgrad |

**OpplæringsprogrammetTrainingTraining program**

Det er ansatt to sykepleiere ved Sentralsykehuset i Vestfold som skal ha hovedansvaret for opplæringsprogrammet. Det vil bli trukket inn undervisere fra sykehuset med spesialkompetanse på de enkelte punktene i undervisningen (anestesi, laboratoriediagnostikk, mikrobiologi, infeksjonsmedisin).Two nurses from The Central Hospital of Vestfold have been recruited to be in charge of the training program. Teachers from the hospital who are specialists in different fields of the education will be recruited (anesthesia, laboratory diagnostics, microbiology, infectious medicine).

The training will be repeated several times so that all relevant personnel have undergone all the theory and training.

1. Opplæring av personell på sykehjem Training of health workers in nursing homes
   1. Teoretisk bakgrunn til alt helsepersonell Theoretical background for all health workers
   2. Praktisk undervisning til alt helsepersonell Hands on training for all health workers
   3. Innhenting av samtykke fra pasienten om innhenting av data Obtaining consent from the patient in regards to collecting data
2. Opplæring av sykehjemsleger Training of nursing home physicians
   1. Teoretisk bakgrunn Theoretical background
   2. Praktisk gjennomføring – spesielt kveld/natt og helg/ferier Practical implementation - especially evening / night and weekend / holidays
3. Opplæring av legevaktsleger Training of emergency physicians
   1. Prosedyre for hva som skal gjøres på sykehjem før henvendelseProcedures for what should be done at a nursing home before inquiry
   2. Momenter i forhold til diagnostikk, intravenøs rehydrering og antibiotikabehandling sykehjem og vurdering behov for av innleggelseElements related to diagnostics, intravenous rehydration and treatment with antibiotic in a nursing home and assessment of the need for hospitalization
4. Kontinuerlig oppfølging over en toårs periode Continuous monitoring over a two-year period
   1. Månedlige besøk på alle sykehjem med oppfrisking av teori og praksisMonthly visits to all nursing homes with a touch up of theory and practice
   2. Mulighet for umiddelbar kontakt med prosjektledelsen ved behovThe possibility for an immediate contact with the project management when needed

**Ethical issues in the intervention**

1. Ethics is included in the education for doctors and nurses to enlarge the consciousness around the legislation and improve the assessment of competence of consent and patient autonomy in life prolonging treatment.
2. Each nursing home will at the time of inclusion be offered aid to reach some kind of minimal level or standard for handling these questions.

Utkommemål og registrering av dataResults Data collection and outcome measures

Each nursing home will have one or more primary contacts (PCs) responsible for registering data from the patient trajectories. Each time a nursing home patient becomes ill and receives treatment with intravenous fluids or intravenous antibiotics – either in the nursing home or in the hospital, specific information will be collected (using study forms/questionnaires). We will compare treatment in the control group (nursing homes before intervention) versus the intervention group and treatment in nursing home versus hospital.

In addition we will perform qualitative components focusing on ethical aspects, as well as satisfaction with the treatment in nursing homes among patients, next-of-kin and the health personnel.

**HelsetjenestenivåHealth service level**

Antall pasienter med behov for intravenøs behandling med væske og/eller antibiotika totalt Antall pasienter med behov for intravenøs behandling som overføres fra sykehjem til sykehusNumber of patients in need of intravenous treatment with fluids and/or antibiotics total Number of patients in need of intravenous treatment that are transferred from a nursing home to a hospital Antall liggedøgn i sykehusNumber of days of hospitalization Utfordringer med den praktiske gjennomføringen av intravenøs behandling i sykehjemmet generelt og ved hvert enkelt sykdomstilfelle (bemanning, kompetanse etc)

**HelseøkonomiHealth Economics**

Kostnader på helsetjenestenivå ved sykehusinnleggelse og ved sykehjemsbehandlingCosts on a health care level at hospitalization and treatment in the nursing home (kost-nytteanalyse under utarbeidelse)(A cost-benefit analysis is under development)

**Pasientnivå Patient Level Antall dager til markert klinisk bedring og til pasienten er i sin habitualtilstand**

Number of days until known clinical improvement and until the patient is back to his/her habitual state Antall dager med behov for intravenøs og påfølgende per oral antibiotikaNumber of days in need for intravenous and succeeding per oral antibiotics Antall dager med behov for intravenøs væskeNumber of days in need of intravenous fluids Antall dager med sengeleieNumber of days of bed rest Direkte og indirekte komplikasjoner til infeksjonDirect and indirect complications related to infection

Acute delirium

Forvirringstilstander kartlagt med strukturert skjemaState of confusion surveyed through a structured form Utfordringer med praktisk gjennomføring av intravenøs behandlingChallenges related to a practical implementation of an intravenous treatment MortalitetMortality

Forekomst av resistente mikrober

**Outcomes realated to intravenous treatment in nursing homes**

Challenges linked to the implementation of intravenous therapy in the nursing home in general and every single illness (manpower, expertise etc)

Challenges with implementing the training program

Tilfredshet hos pasienter, pårørende og hos sykehjemspersonelletSatisfaction among patients, next of kin and in nursing personnel (Strukturert spørreskjemaundersøkelseStructured survey and qualitative interviews) Kvalitative intervjuer

**Ethical issues**

1. Qualitative interviews with doctors, nurses, next of kin to assess ethical and practical challenges concering
   1. Assessment of consent competence
   2. Preparatory conversations
   3. Decision process concerning life prolonging treatment by acute disease or deterioration
2. A quantitative investigation with a questionnaire filled in for I) Patient getting an infection or a dehydration period where treatment is chosen not to be given II) Patients included in the main study getting treatment for an infection or a dehydration episode. Tentative questions:
   1. Is the assessment of consent competence done and documented?
   2. Is a conversation of treatment carried through or documented?
   3. If no, why not?
   4. If yes, who participated?
   5. Ethical dilemmas?
   6. Practical challenges?

Materiale**Power calculation**

Intervensjonen skal gjennomføres på 28 sykehjem med om lag 1 400 beboere.The intervention will be carried out in 34 nursing homes inhabiting around 1 500 residents. Antatt årlig forekomst av behov for intravenøs væske: 10%?Expected yearly incidences of need for intravenous fluids: 800 patients Antatt årlig forekomst av behov for intravenøs antibiotika: 5%?Expected yearly incidences of need for intravenous fluids: 400 patients

Expected yearly incidences of need for intravenous antibiotics: 200 patients

Our power calculation is based on a reduction of hospital admissions of patients in need of intravenous antibiotics from 90% to 65%, or 25%. Furthermore we assume an alfa 0.05 and beta 0.80. We assume a cluster-coefficient of 0.10. The calculation gives an estimate of 56 patients in each group, or 4 patients per nursing home on average (n=28) With a drop out proportion of 10%, this is increased to 65 patients in each group with a total of 130 patients, or 5 patients per nursing home on average.

Time schedule

| **2009** | **2010 og 2011** | **2012** |
| --- | --- | --- |
| Compute protocol and research forms  Apply REK and Clinical Trials  Apply for funds  **November 2009: Intervention start** | Gjennomføring av intervensjon  Datainnsamling og databearbeiding  Project meetings (nursing home contacts, doctors and leaders)  **Autumn 2011: Intervention ends** | Data analysis  Writing of reports  Writing of research papers  **Autumn 2012: Project ends** |

Management, organization and affiliated institutions

- Universitetet i Oslo ved Maria Romøren og Morten LindbækThe University of Oslo represented by Maria Romøren, Morten Lindbæk and Lisbeth Østby
- NTNU ved Helge GaråsenNTNU represented by Helge Garåsen
- Intravenøs-prosjektet i Telemark ved Lisbeth ØstbyThe intravenous project in Telemark represented by Lisbeth Østby
- The Undervisningssykehjemmet.Teaching nursing home ini Vestfold ved Vibeke Bostrøm og Bjørn Schreiner Vestfold represented by Vibeke Bostrøm and Bjørn Schreiner
- Sykehuset i Vestfold ved Irene Jørgensen, Halvdan Aass, Lars Rustad og Per BjarkVestfold Hospital represented by Irene Jørgensen, Halfdan Aass, Lars Rustad and Per BjarkHøyskolen i Vestfold ved Solveig Osland og May Elin Thengs Horntvedt.
- Vestfold University College represented by Solveig Osland and May Elin Thengs HorntvedtLeder for sykehjemmene i Larvik Inger Lund Thorsen
- Head of the nursing home in Larvik, Inger Lund Thorsen
- Alle sykehjem i VestfoldAll nursing homes in Vestfold

LitteraturLiterature

Andersen B M., Rasch M. Nosokomiale infeksjoner i sykehjem i Oslo. Andersen B M., Rasch M. Nosocomiale infections in nursing home in Oslo. Tidsskr Nor Legeforen 2002; 122:2371-2373. Osteoporosis Foren 2002; 122:2371-2373.

Boockvar KS, Gruber-Baldini AL, Burton L, Zimmermann S, May C, Magaziner J. Outcomes of infection in nursing home residents with and without early hospital transfer. J Am Geriatr  Soc 2005; 53: 590-6. Boockvar KS, Gruber-Baldini AL, Burton L, Zimmerman S, May C, Magazine J. Outcomes of infection in nursing home residents with and without early hospital transfer. J Am Geriatr Soc 2005; 53: 590-6.

Dosa D. [Should I hospitalize my resident with nursing home-acquired pneumonia?](http://translate.google.com/translate?hl=no&sl=no&tl=en&prev=hp&u=http://linkinghub.elsevier.com/retrieve/pii/S1525-8610(05)00387-7) . J Am Med Dir Assoc 6:327–33. Dosa D. [Should I hospitalize my resident with nursing home-acquired pneumonia?.](http://translate.google.com/translate?hl=no&sl=no&tl=en&prev=hp&u=http://linkinghub.elsevier.com/retrieve/pii/S1525-8610(05)00387-7) J Am Med Dir Assoc 6:327-33.

Fried TR, Gillick MR, Libsitz LA. Fried TR, Gillick MR, Libsitz LA. Short-term functional outcomes of long-term care residents with pneumonia treated with and without hospital transfer. Short-term functional outcomes of long-term care residents with pneumonia treated with and without hospital transfer. . . J Am Geriatr  Soc 1997;45: 302-6. J Am Geriatr Soc 1997; 45: 302-6.

Fried TR, Gillick MR, Libsitz LA. Fried TR, Gillick MR, Libsitz LA. Whether to transfer? Whether two transfer? Factors associated with hospitalization and ouotcome of elderly long-term care patients with pneumonia. Factors associated with hospitalization and ouotcome of elderly long-term care patients with pneumonia. J  Gen Intern Med 1995; 10: 246-50. J Gen Intern Med 1995; 10: 246-50.

Høie J. Finanisering av legetjenester i sykehjem. Høie J. funding of medical services in nursing homes. Tidsskr Nor Legeforen 2009; 124: 85. Osteoporosis Foren 2009; 124: 85

Kielv *et al* . Kielv *et al.* Persistent delirium predicts greater mortality. Persistent delirium predicts greater mortality. JAGS 2009. JAGS 2009.

Mark Loeb, Kevin Brazil, Lynne Lohfeld, Allison McGeer, Andrew Simor, Kurt Stevenson, Dick Zoutman, Stephanie Smith, Xiwu Liu, Stephen D Walter. Mark Loeb, Kevin Brazil, Lynne Lohfelden, Allison McGeer, Andrew Simor, Kurt Stevenson, Dick Zoutman, Stephanie Smith, Xiwu Liu, Stephen D Walter. Effect of a multifaceted intervention on number of antimicrobial prescriptions for suspected urinary tract infections in residents of nursing homes: cluster randomised controlled trial **.** BMJ Sep 2005; 331: 669;doi:10.1136/bmj.38602.586343.55 Effect of a multifaceted intervention Wed number of antimicrobial prescriptions for suspected urinary tract infections in residents of nursing homes: cluster randomized controlled **trial.** BMJ September 2005; 331: 669, doi: 10.1136/bmj.38602.586343.55

Medical Research Council UK.Developing and evaluating complex interventions: new guidance: 2006. www.mrc.ac.uk/complexinterventionsguidance

Nygaard HA, Naik M, Ruths S. Mental svikt hos sykehjemspasienter. Nygaard HA, Naik M, Ruths S. Mental failure at nursing home patients. Tidsskr Nor Legeforen 2000; 120: Osteoporosis Foren 2000; 120:

Ranhoff AH, Linnsund JM: Når skal sykehjemspasienter innlegges i sykehus? Ranhoff AH, Linn Sund JM: When should nursing home patients admitted to hospital? Tidsskr Nor Lægeforen 2005;125:1844-7 Tidsskr Nor Laegeforen 2005, 125:1844-7

Sayers GM, Perera S. Withholding life prolonging treatment, and self deception. Sayers GM, Perera S. withholding life prolonging treatment, and self deception. J Med Ethics 2002;28:347-52. J Med Ethics 2002; 28:347-52.

Sheldon P Stone, CC Kibbler, C Bowman, and David Stott Controlling infection in British nursing homes. BMJ Mar 2001; 322: 506; doi:10.1136/bmj.322.7285.506 Sheldon P Stone, CC Kibble, C Bowman, and David Stott Controlling infection in British nursing homes. BMJ March 2001: 322: 506, doi: 10.1136/bmj.322.7285.506

Helling L. Sykehjem og medisinsk behandling. Slope L. Nursing home and medical treatment. Tidsskr Nor Legeforen 2008; 128:843. Osteoporosis Foren 2008; 128:843.

Helling L. Sykehjem som behandlingsinstitusjon. Tidsskr Nor Lægeforen 2007; 127:2277. Slope L. Nursing home as a treatment institution. JNMA 2007; 127:2277.

Tobiassen T. Berild D. Hjortdahl P. Tobiassen T. Berild D. Hjortdahl P. Bruk av systemiske antibiotika ved norske sykehjem. Use of systemic antibiotics in Norwegian nursing homes. Tidsskr Nor Legeforen  2002; 122:2376-2378. Osteoporosis Foren 2002; 122:2376-2378.

Van der Steen JT, Kruse RL, Ooms ME, Ribbe MW, van der Wal G, heintz LL, Mehr DR. Van der Steen JT, Kruse RL, Ooms ME, Wall MW, van der Wal G, Heintz LL, Mehr DR. Treatment of nursing home residents with dementia and lower respiratory tract infection in the United States and The Netherlands: an ocean apart. Treatment of nursing home residents with dementia and lower respiratory tract infection in the United States and The Netherlands: an ocean apart. J Am Geriatr  Soc 2004; 23: 691-9. J Am Geriatr Soc 2004; 23: 691-9.
