# Supplementary material for: A structured training program for health workers in intravenous treatment with fluids and antibiotics in nursing homes: A modified stepped-wedge cluster-randomised trial to reduce hospital admissions
Source: PLoS One. 2017 Sep 7;12(9):e0182619. doi: 10.1371/journal.pone.0182619 (PMC5589147; doi:10.1371/journal.pone.0182619)
Supplement: S2 Appendix — Basic form for all patients. (DOC) [file pone.0182619.s004.doc]

| Navn __________________________Født ____________ | | **Skjema 1 – Felles pasientinformasjon s1** | |
| --- | --- | --- | --- |
| Kjønn  K  M | |  | |
| Sykehjem ___________________________________ | Type plass  Rehabilitering   Korttidsavdeling   Langtidsavdeling   Kombinert kort- og langtidsavdeling   Demensenhet/skjermet enhet   Palliativ enhet | |  |

| Første undersøkelse SYKEPLEIER | Dato  (ddmmåå) |
| --- | --- |

| **Nytilkomne upesifikke symptomer/funn** | | **Urinveier**  Ubehaglig/hyppig vannlatning  Ny/økende urininkontinens/retensjon  Endret lukt/farve eller uklar urin  Urin stix  *Leukocytter____ Nitritt____ Blod____*  **Luftveier**  Surklete respirasjon  Hoste  **Hud/bløtdel**  Sår med puss/belegg  Rødhet  Varme  Hevelse  Lokalisert smerte  **Dehydrering**  Tørr hud  Stående hudfolder  Mindre urinproduksjon  Blodprøver  *Hb ___.__ B-glukose___.__* |  Nei  Ja   Nei  Ja   Nei  Ja   Nei  Ja   Nei  Ja   Nei  Ja   Nei  Ja   Nei  Ja   Nei  Ja   Nei  Ja   Nei  Ja   Nei  Ja   Nei  Ja   Nei  Ja   Nei  Ja   Nei  Ja |
| --- | --- | --- | --- |
| Feber  Feber m/frostanfall  Redusert allmenntilstand  Ubehagelig/surklete respirasjon  Kvalme/brekninger  Mage og/eller ryggsmerter  Fall/falltendens  Annet _________________________  ______________________________ |  Nei  Ja   Nei  Ja   Nei  Ja   Nei  Ja   Nei  Ja   Nei  Ja   Nei  Ja   Nei  Ja |

Klinisk status dag 1

| BT ____/____ Puls _____ Temp ___.__ Respirasjonsfrekvens____ CRP ____  Bevissthet  Våken  Somnolent  Bevisstløs  Matinntak  Normalt  Redusert  Sonde  Væskeinntak  Normalt  Redusert  Intravenøs væske |
| --- |

|  Confusion Assessment Method (CAM) fylt ut (s 4)   Barthel ADL-Index fylt ut (s 5)   Kopi av medikamentliste vedlagt | |
| --- | --- |
| Navn:________________________Født:____________ | **Skjema 1 – Felles pasientinformasjon s2** |

| Konklusjon første undersøkelse LEGE | Dato  (ddmmåå) |
| --- | --- |

| **Type konsultasjon** | 1.  2. |  Legevaktslege   Telefonkonsultasjon |  Fast sykehjemslege   Klinisk undersøkelse |
| --- | --- | --- | --- |

| **Tentativ diagnose**  (en/flere) |  Dehydrering   Øvre luftveisinfeksjon   Nedre luftveisinfeksjon   Forverring av KOLS eller kronisk bronkitt   Nedre urinveisinfeksjon   Øvre urinveisinfeksjon   Overfladisk hudinfeksjon, infisert diabetisk fotsår etc.   Dyp hudinfeksjon   Overfladisk postoperativ sårinfeksjon   Dyp postoperativ sårinfeksjon   Annet: ______________________________________________________   Usikkert: |
| --- | --- |

Tiltak

 Kun lindrende behandling på sykehjemmet (ikke antibiotika eller intravenøs væske)

 Behandling på sykehjem

|  Peroral antibiotika | **Skjema 2 – PO/IM ANTIBIOTIKA** |
| --- | --- |

|  Kun intravenøs væske | **Skjema 3 – IV VÆSKE** |
| --- | --- |

|  Intravenøs antibiotika | **Skjema 4 – IV ANTIBIOTIKA** |
| --- | --- |

 Sykehusinnleggelse

|  Antatt behov for i.v. behandling - sykehjem ikke fått opplæring   Antatt behov for i.v. behandling - sykehjem har ikke kapasitet/kompetanse akkurat nå  Beskriv nærmere_______________________________________________________________________  _____________________________________________________________________________________  _____________________________________________________________________________________   Intravenøs behandling på sykehjem ikke aktuelt – på grunn av  1.  Kompliserende faktorer/komorbiditet  2.  Alvorlig infeksjon i buken  3. ** Alvorlighetsgrad/sepsisutvikling (innleggelse vurderes hvis tre eller flere kriterier er oppfylt)  Temperatur >38.5 eller <35.0  Puls >100/min  Respirasjon >30/min  Systolisk BT < 90 mmHg  Oksygenmetning < 92%   Behov for diagnostisk avklaring, kan komme tilbake for intravenøs behandling |
| --- |

| Navn:________________________Født:____________ | **Skjema 1 – Felles pasientinformasjon s3** |
| --- | --- |

| Bakgrunnsinformasjon Fylles ut av sykehjemslege |
| --- |

| Kjente grunnsykdommer | |  |
| --- | --- | --- |
|  | KOLS  Angina/tidligere hjerteinfarkt  Atrieflimmer  Hjertesvikt  Demens  Hjerneslag  Diabetes  Kreftsykdom (hvilken?):______________________________________  Fot/leggsår  Liggesår/trykksår  Redusert perifer sirkulasjon  Annet:_____________________________________________________ |  Nei  Ja   Nei  Ja   Nei  Ja   Nei  Ja   Nei  Ja   Nei  Ja   Nei  Ja   Nei  Ja   Nei  Ja   Nei  Ja   Nei  Ja   Nei  Ja |
| Risiko for urinveisinfeksjon | |  |
|  | Inkontinent for urin, bruker bleier  Intermitterende urinkateterisering  Permanent urinkateter |  Nei  Ja   Nei  Ja   Nei  Ja |
| Risiko for sykehusrelatert infeksjon | |  |
|  | Fra sykehus < 48 t  Operert siste 30 dager  Operert inn fremmedlegeme siste 12 mnd |  Nei  Ja   Nei  Ja   Nei  Ja |

| **Dag 1** Confusion Assessment Method (CAM) | **Skjema 1 – Felles pasientinformasjon s4** |
| --- | --- |

| Inouye et al. Ann Int Med 1990; 113: 941-948.  Norsk oversettelse ved Anette Hylen Ranhoff, Marianne Hjermstad og Jon Håvard Loge, 2004. |
| --- |

**GENERELT**

Delirium (tidligere ofte kalt akutt forvirring eller akutt konfusjon) er en vanlig komplikasjon til akutt sykdom hos gamle. Det finnes flere typer, pasientene kan bli enten hyperaktive (agiterte), hypoaktive (stille), eller veksler mellom disse. Alvorlighetsgraden kan variere betydelig.

Delirium krever rask diagnostikk og intervensjon. Confusion Assessment Method (CAM) er en kort screeningtest som gir diagnosen med god presisjon (basert på DSM-III og ICD-10). Algoritmen er velegnet for påvisning og oppfølging av delirium i klinisk praksis. Spørsmålene skal besvares av helsepersonell og baseres på egen kjennskap til pasienten eller samtale med personale eller pårørende som kjenner vedkommende.

| Barthel ADL-Indeks (status for 14 dager siden) | **Skjema 1 – Felles pasientinformasjon s5** |
| --- | --- |

| Mahoney FI, Barthel DW. Maryland State Med J 1965;14:61-65.  Denne norske versjonen er redigert i 2008 av Ingvild Saltvedt, Jorunn L. Helbostad, Unni Sveen, Pernille Thingstad, Olav Sletvold  og Torgeir Bruun Wyller på grunnlag av flere tidligere norske oversettelser og med hovedvekt på originalpublikasjonen fra 1965. |
| --- |

**GENERELT**

Barthel ADL-indeks er først og fremst beregnet på å bli brukt av sykepleiere, ergoterapeuter og fysioterapeuter i deres daglige kontakt med pasientene. Det skal registreres hva pasienten faktisk gjør, ikke hva man tror pasienten kan mestre. Svarene skal baseres på egen kjennskap til pasienten eller samtale med personale eller pårørende som kjenner vedkommende. Pasienten skal ikke ”testes”. Poengene representerer grad av uavhengighet av hjelp fra annen person, uansett årsak. Hvis det er nødvendig med *tilsyn* eller *tilrettelegging*, er personen **ikke** uavhengig, men hvis en aktivitet mestres med *hjelpemidler,* **er** personen uavhengig i denne aktiviteten.
