# Supplementary material for: A structured training program for health workers in intravenous treatment with fluids and antibiotics in nursing homes: A modified stepped-wedge cluster-randomised trial to reduce hospital admissions
Source: PLoS One. 2017 Sep 7;12(9):e0182619. doi: 10.1371/journal.pone.0182619 (PMC5589147; doi:10.1371/journal.pone.0182619)
Supplement: S3 Appendix — Form for patients treated with iv antibiotics in hospital. (DOC) [file pone.0182619.s005.doc]

| Navn:________________________Født:____________ | **Sykehusskjema – IV ANTIBIOTIKA s1** | | | |
| --- | --- | --- | --- | --- |
| Kjønn  K  M | | |  | |
| Avdeling  Geriatrisk avdeling   Hjerteavdelingen   Infeksjonsmedisin   Hematologisk avdeling   Gastro (medisin)   Lungeseksjonen   Nyreseksjonen   Kirurgisk avdeling   Annen: _______________________ | | Innleggelsestidspunkt  Dato  (ddmmåå) Kl _ _ . _ _  **Innleggelsesdiagnose/problemstilling** (en/flere)  1. _________________________________________  2. _________________________________________  3. _________________________________________ | |  |

| **Diagnose**  (en/flere) |  Pneumoni   Øvre urinveisinfeksjon |  Dyp hudinfeksjon   Postoperativ sårinfeksjon |  Usikkert __________________   Annet ____________________ |
| --- | --- | --- | --- |

Akuttbehandling

| Dato | Tidspunkt første dose | Medikament  (produktnavn) | Døgndose  (mg/g/IE) | Dosering  (mg/g/IE)x__ | Seponert dato | Behandlings svikt? (ja/nei) | Antall dagers  behandling |
| --- | --- | --- | --- | --- | --- | --- | --- |
|  |  |  |  |  |  |  |  |
|  |  |  |  |  |  |  |  |
|  |  |  |  |  |  |  |  |
|  |  |  |  |  |  |  |  |
|  |  |  |  |  |  |  |  |
|  |  |  |  |  |  |  |  |
|  |  |  |  |  |  |  |  |

Seponerte medikamenter: ____________________________________________________________________

Annen iverksatt behandling: __________________________________________________________________

_________________________________________________________________________________________

Klinisk status dag 1

| BT ____/____ Puls _____ Temp ___.__ Respirasjonsfrekvens____ CRP ____  Bevissthet  Våken  Somnolent  Bevisstløs  Matinntak  Normalt  Redusert  Sonde  Væskeinntak  Normalt  Redusert  Intravenøs væske |
| --- |

|  Skjema om beslutningsprosessen fylt ut (s 6)   Confusion Assessment Method (CAM) fylt ut for dag 1 (s 7)   Kopi av medikamentliste dag 1 vedlagt |
| --- |

| Navn:________________________Født:____________ | **Sykehusskjema – IV ANTIBIOTIKA s2** |
| --- | --- |

Klinisk status dag 2

| BT ____/____ Puls _____ Temp ___.__ Respirasjonsfrekvens____ CRP ____ (hvis tatt)  Bevissthet  Våken  Somnolent  Bevisstløs  *Fylles ut hos de pasientene som hadde avvikende resultater dag 1* |
| --- |

|  Confusion Assessment Method (CAM) fylt ut for dag 2 (s 8) |
| --- |

Viktige merknader (f. eks. komplikasjoner)_______________________________________________________________

_________________________________________________________________________________________________

_________________________________________________________________________________________________

Klinisk status dag 3

| BT ____/____ Puls _____ Temp ___.__ Respirasjonsfrekvens____ CRP ____ (hvis tatt)  Bevissthet  Våken  Somnolent  Bevisstløs  *Fylles ut hos de pasientene som hadde avvikende resultater dag 2* |
| --- |

|  Confusion Assessment Method (CAM) fylt ut for dag 3 (s 9) |
| --- |

Viktige merknader (f. eks. komplikasjoner)_______________________________________________________________

_________________________________________________________________________________________________

_________________________________________________________________________________________________

Klinisk status dag 4

| BT ____/____ Puls _____ Temp ___.__ Respirasjonsfrekvens____ CRP ____ (hvis tatt)  Bevissthet  Våken  Somnolent  Bevisstløs  *Fylles ut hos de pasientene som hadde avvikende resultater dag 3* |
| --- |

|  Confusion Assessment Method (CAM) fylt ut for dag 4 (s 10) |
| --- |

Viktige merknader (f. eks. komplikasjoner)_______________________________________________________________

_________________________________________________________________________________________________

_________________________________________________________________________________________________

Klinisk status dag 5

| BT ____/____ Puls _____ Temp ___.__ Respirasjonsfrekvens____ CRP ____ (hvis tatt)  Bevissthet  Våken  Somnolent  Bevisstløs  *Fylles ut hos de pasientene som hadde avvikende resultater dag 4* |
| --- |

|  Confusion Assessment Method (CAM) fylt ut for dag 5 (s 11) |
| --- |

Viktige merknader (f. eks. komplikasjoner)_______________________________________________________________

_________________________________________________________________________________________________

_________________________________________________________________________________________________

| Navn:________________________Født:____________ | **Sykehusskjema – IV ANTIBIOTIKA s3** |
| --- | --- |

Klinisk status dag 7

| BT ____/____ Puls _____ Temp ___.__ Respirasjonsfrekvens____ CRP ____ (hvis tatt)  Bevissthet  Våken  Somnolent  Bevisstløs  *Fylles ut hos de pasientene som hadde avvikende resultater dag 5* |
| --- |

|  Confusion Assessment Method (CAM) fylt ut for dag 7 (s 12) |
| --- |

Viktige merknader (f. eks. komplikasjoner)_______________________________________________________________

_________________________________________________________________________________________________

_________________________________________________________________________________________________

Klinisk status dag 10

| BT ____/____ Puls _____ Temp ___.__ Respirasjonsfrekvens____ CRP ____ (hvis tatt)  Bevissthet  Våken  Somnolent  Bevisstløs  *Fylles ut hos de pasientene som hadde avvikende resultater dag 7* |
| --- |

|  Confusion Assessment Method (CAM) fylt ut for dag 10 (s 13) |
| --- |

Viktige merknader (f. eks. komplikasjoner)_______________________________________________________________

_________________________________________________________________________________________________

_________________________________________________________________________________________________

Klinisk status dag 15

| BT ____/____ Puls _____ Temp ___.__ Respirasjonsfrekvens____ CRP ____ (hvis tatt)  Bevissthet  Våken  Somnolent  Bevisstløs  *Fylles ut hos de pasientene som hadde avvikende resultater dag 10* |
| --- |

|  Confusion Assessment Method (CAM) fylt ut for dag 15 (s 14) |
| --- |

Viktige merknader (f. eks. komplikasjoner)_______________________________________________________________

_________________________________________________________________________________________________

_________________________________________________________________________________________________

Klinisk status dag 30

| BT ____/____ Puls _____ Temp ___.__ Respirasjonsfrekvens____ CRP ____ (hvis tatt)  Bevissthet  Våken  Somnolent  Bevisstløs  *Fylles ut hos de pasientene som hadde avvikende resultater dag 15* |
| --- |

|  Confusion Assessment Method (CAM) fylt ut for dag 30 (s 15) |
| --- |

Viktige merknader (f. eks. komplikasjoner)_______________________________________________________________

_________________________________________________________________________________________________

_________________________________________________________________________________________________

| Navn:________________________Født:____________ | **Sykehusskjema – IV ANTIBIOTIKA s4** |
| --- | --- |

Oppsummering ved utskrivelse (Utskrivelsesdato  ddmmåå)

| **Sykdomsforløp**  ___ dager med intravenøs væske  ___ dager før klinisk frisk  Tilbake i normaltilstand?  Ja  Nei  Kjenner ikke pasientens normaltilstand   Død ___ dager etter oppstart av behandling | | |
| --- | --- | --- |
| **Komplikasjoner**  Reaksjon på antibiotika    Liggesår  Fall med skade  Delirium påvist med CAM  Sykehusinfeksjon  Annet |  Nei  Ja   Nei  Ja:   Nei  Ja:   Nei  Ja   Nei  Ja:   Nei  Ja: |  Kvalme/oppkast  Hudutslett   Sjokk  ________________________________________  ________________________________________  ___ dager  ________________________________________  ________________________________________ |
| **Intravenøs behandling**  Komplikasjoner ved intravenøs behandling (infeksjon i venen, hematom, utstyrssvikt el.l.)  Nei  Ja:______  _________________________________________________________________________________________  Utfordringer/ulemper ved sykehusbehandling av denne pasienten: ___________________________________  _________________________________________________________________________________________  _________________________________________________________________________________________  Fordeler med sykehusbehandling av denne pasienten: ______________________________________________  _________________________________________________________________________________________  _________________________________________________________________________________________ | | |

| Navn:________________________Født:____________ | **Sykehusskjema – IV ANTIBIOTIKA s5** |
| --- | --- |

Oppsummering 30 dager etter debut av aktuelle sykdom

| **Sykdomsforløp**  ___ dager med intravenøs væske  ___ dager før klinisk frisk  Tilbake i normaltilstand?  Ja  Nei  Kjenner ikke pasientens normaltilstand   Død ___ dager etter oppstart av behandling | | |
| --- | --- | --- |
| **Komplikasjoner**  Reaksjon på antibiotika    Liggesår  Fall med skade  Delir  Sykehusinfeksjon  Annet |  Nei  Ja   Nei  Ja:   Nei  Ja:   Nei  Ja   Nei  Ja:   Nei  Ja: |  Kvalme/oppkast  Hudutslett   Sjokk  ________________________________________  ________________________________________  ___ dager  ________________________________________  ________________________________________ |
| **Intravenøs behandling**  Komplikasjoner ved intravenøs behandling (infeksjon i venen, hematom, utstyrssvikt el.l.)  Nei  Ja:______  _________________________________________________________________________________________  Utfordringer/ulemper ved sykehusbehandling av denne pasienten sett fra sykehjemmets side:_______________  _________________________________________________________________________________________  _________________________________________________________________________________________  Fordeler med sykehusbehandling av denne pasienten sett fra sykehjemmets side: ________________________  _________________________________________________________________________________________  _________________________________________________________________________________________ | | |

Huskeliste 30 dager etter debut av aktuelle sykdom

|  Sykehusinfeksjon registrert (debut på sykehus/<48 t etter utskrivelse)   Nei  Ja: ____________________________________________________   Samtykke-erklæring vedlagt (pasientens underskrift på deltakelse i studien)   Barthel ADL-Index fylt ut – for situasjonen 30 dager etter sykdomsdebut (s 16)   Kopi av dagens medikamentliste vedlagt   Pasienten er skrevet ut til hjemmet. Ring Lisbeth Østby, 91820728   Pasienten er død ____ dager etter oppstart av behandling |
| --- |

| Navn:________________________Født:____________ | **Sykehusskjema – IV ANTIBIOTIKA s6** |
| --- | --- |

Beslutningsprosessen - Fylles ut av sykehuslege på avdelingen, helst behandlingsansvarlig lege

| 1. Var det under oppholdet noen gang tvil om denne pasienten burde vært innlagt?  Ja  Nei  Hvis ja, hva var grunnen til det (ett eller flere kryss)?   Pasienten kunne fått behandling for tilstanden på sykehjemmet   Tvil om fordelene ved innleggelse for pasienten var større enn ulempene   Tvil om livsforlengende behandling var riktig for denne pasienten   Annet _________________________________________________________________  2. Var det noen gang tvil om intravenøs behandling var riktig for denne pasienten?  Ja  Nei  Hvis ja, var det en eller flere av de følgende grunnene?   Tvil om behandlingen var til pasientens beste   Tvil om pasienten faktisk ønsket intravenøs behandling   Tvil om hva slags behandling pårørende ønsket at pasienten skulle få   Tvil om behandlingen ville gi effekt   Det var uenighet i behandlingsteamet om behandlingen   Annet ________________________________________________________________  3. Ble behandlingen diskutert med sykehjemspersonell som kjenner pasienten?  Ja  Nei  Vet ikke  Hvis nei, hvorfor ikke? ________________________________________________________________  4. Ble behandlingen på sykehuset diskutert med pasienten før oppstart?  Ja  Nei  Vet ikke  Hvis nei, hvorfor ikke? ________________________________________________________________  5. Ble behandlingen på sykehuset diskutert med pårørende før oppstart ?  Ja  Nei  Vet ikke  Hvis nei, hvorfor ikke? ________________________________________________________________  6. Ble pasientens samtykkekompetanse vurdert på sykehuset før behandlingen?  Ja  Nei  Vet ikke  Hvis ja, var pasienten samtykkekompetent?  Ja  Nei  Hvis nei, hvorfor ble samtykkekompetanse ikke vurdert (gjerne flere kryss)?   Pasienten var helt opplagt samtykkekompetent   Pasienten var helt opplagt ikke samtykkekompetent   Vi hadde ikke tid til å vurdere det   Usikkerhet i forhold til hvordan en vurderer samtykkekompetanse   Annet _________________________________________________________________  7. Er det *tidligere* (på sykehjem eller på sykehus) gjennomført samtaler med pasient eller pårørende om pasientens ønsker og verdier i forhold til livsforlengende behandling eller hva som skal gjøres ved akutt forverring av pasientens helsetilstand?  Ja  Nei  Vet ikke |
| --- |

| **Dag 1** Confusion Assessment Method (CAM) | **Sykehusskjema – IV ANTIBIOTIKA s7** |
| --- | --- |

| Inouye et al. Ann Int Med 1990; 113: 941-948.  Norsk oversettelse ved Anette Hylen Ranhoff, Marianne Hjermstad og Jon Håvard Loge, 2004. |
| --- |

**GENERELT**

Delirium (tidligere ofte kalt akutt forvirring eller akutt konfusjon) er en vanlig komplikasjon til akutt sykdom hos gamle. Det finnes flere typer, pasientene kan bli enten hyperaktive (agiterte), hypoaktive (stille), eller veksler mellom disse. Alvorlighetsgraden kan variere betydelig.

Delirium krever rask diagnostikk og intervensjon. Confusion Assessment Method (CAM) er en kort screeningtest som gir diagnosen med god presisjon (basert på DSM-III og ICD-10). Algoritmen er velegnet for påvisning og oppfølging av delirium i klinisk praksis. Spørsmålene skal besvares av helsepersonell og baseres på egen kjennskap til pasienten eller samtale med personale eller pårørende som kjenner vedkommende.

| **Dag 2** Confusion Assessment Method (CAM) | **Sykehusskjema – IV ANTIBIOTIKA s8** |
| --- | --- |

| Inouye et al. Ann Int Med 1990; 113: 941-948.  Norsk oversettelse ved Anette Hylen Ranhoff, Marianne Hjermstad og Jon Håvard Loge, 2004. |
| --- |

**GENERELT**

Delirium (tidligere ofte kalt akutt forvirring eller akutt konfusjon) er en vanlig komplikasjon til akutt sykdom hos gamle. Det finnes flere typer, pasientene kan bli enten hyperaktive (agiterte), hypoaktive (stille), eller veksler mellom disse. Alvorlighetsgraden kan variere betydelig.

Delirium krever rask diagnostikk og intervensjon. Confusion Assessment Method (CAM) er en kort screeningtest som gir diagnosen med god presisjon (basert på DSM-III og ICD-10). Algoritmen er velegnet for påvisning og oppfølging av delirium i klinisk praksis. Spørsmålene skal besvares av helsepersonell og baseres på egen kjennskap til pasienten eller samtale med personale eller pårørende som kjenner vedkommende.

| **Dag 3** Confusion Assessment Method (CAM) | **Sykehusskjema – IV ANTIBIOTIKA s9** |
| --- | --- |

| Inouye et al. Ann Int Med 1990; 113: 941-948.  Norsk oversettelse ved Anette Hylen Ranhoff, Marianne Hjermstad og Jon Håvard Loge, 2004. |
| --- |

**GENERELT**

Delirium (tidligere ofte kalt akutt forvirring eller akutt konfusjon) er en vanlig komplikasjon til akutt sykdom hos gamle. Det finnes flere typer, pasientene kan bli enten hyperaktive (agiterte), hypoaktive (stille), eller veksler mellom disse. Alvorlighetsgraden kan variere betydelig.

Delirium krever rask diagnostikk og intervensjon. Confusion Assessment Method (CAM) er en kort screeningtest som gir diagnosen med god presisjon (basert på DSM-III og ICD-10). Algoritmen er velegnet for påvisning og oppfølging av delirium i klinisk praksis. Spørsmålene skal besvares av helsepersonell og baseres på egen kjennskap til pasienten eller samtale med personale eller pårørende som kjenner vedkommende.

| **Dag 4** Confusion Assessment Method (CAM) | **Sykehusskjema – IV ANTIBIOTIKA s10** |
| --- | --- |

| Inouye et al. Ann Int Med 1990; 113: 941-948.  Norsk oversettelse ved Anette Hylen Ranhoff, Marianne Hjermstad og Jon Håvard Loge, 2004. |
| --- |

**GENERELT**

Delirium (tidligere ofte kalt akutt forvirring eller akutt konfusjon) er en vanlig komplikasjon til akutt sykdom hos gamle. Det finnes flere typer, pasientene kan bli enten hyperaktive (agiterte), hypoaktive (stille), eller veksler mellom disse. Alvorlighetsgraden kan variere betydelig.

Delirium krever rask diagnostikk og intervensjon. Confusion Assessment Method (CAM) er en kort screeningtest som gir diagnosen med god presisjon (basert på DSM-III og ICD-10). Algoritmen er velegnet for påvisning og oppfølging av delirium i klinisk praksis. Spørsmålene skal besvares av helsepersonell og baseres på egen kjennskap til pasienten eller samtale med personale eller pårørende som kjenner vedkommende.

| **Dag 5** Confusion Assessment Method (CAM) | **Sykehusskjema – IV ANTIBIOTIKA s11** |
| --- | --- |

| Inouye et al. Ann Int Med 1990; 113: 941-948.  Norsk oversettelse ved Anette Hylen Ranhoff, Marianne Hjermstad og Jon Håvard Loge, 2004. |
| --- |

**GENERELT**

Delirium (tidligere ofte kalt akutt forvirring eller akutt konfusjon) er en vanlig komplikasjon til akutt sykdom hos gamle. Det finnes flere typer, pasientene kan bli enten hyperaktive (agiterte), hypoaktive (stille), eller veksler mellom disse. Alvorlighetsgraden kan variere betydelig.

Delirium krever rask diagnostikk og intervensjon. Confusion Assessment Method (CAM) er en kort screeningtest som gir diagnosen med god presisjon (basert på DSM-III og ICD-10). Algoritmen er velegnet for påvisning og oppfølging av delirium i klinisk praksis. Spørsmålene skal besvares av helsepersonell og baseres på egen kjennskap til pasienten eller samtale med personale eller pårørende som kjenner vedkommende.

| **Dag 7** Confusion Assessment Method (CAM) | **Sykehusskjema – IV ANTIBIOTIKA s12** |
| --- | --- |

| Inouye et al. Ann Int Med 1990; 113: 941-948.  Norsk oversettelse ved Anette Hylen Ranhoff, Marianne Hjermstad og Jon Håvard Loge, 2004. |
| --- |

**GENERELT**

Delirium (tidligere ofte kalt akutt forvirring eller akutt konfusjon) er en vanlig komplikasjon til akutt sykdom hos gamle. Det finnes flere typer, pasientene kan bli enten hyperaktive (agiterte), hypoaktive (stille), eller veksler mellom disse. Alvorlighetsgraden kan variere betydelig.

Delirium krever rask diagnostikk og intervensjon. Confusion Assessment Method (CAM) er en kort screeningtest som gir diagnosen med god presisjon (basert på DSM-III og ICD-10). Algoritmen er velegnet for påvisning og oppfølging av delirium i klinisk praksis. Spørsmålene skal besvares av helsepersonell og baseres på egen kjennskap til pasienten eller samtale med personale eller pårørende som kjenner vedkommende.

| **Dag 10** Confusion Assessment Method (CAM) | **Sykehusskjema – IV ANTIBIOTIKA s13** |
| --- | --- |

| Inouye et al. Ann Int Med 1990; 113: 941-948.  Norsk oversettelse ved Anette Hylen Ranhoff, Marianne Hjermstad og Jon Håvard Loge, 2004. |
| --- |

**GENERELT**

Delirium (tidligere ofte kalt akutt forvirring eller akutt konfusjon) er en vanlig komplikasjon til akutt sykdom hos gamle. Det finnes flere typer, pasientene kan bli enten hyperaktive (agiterte), hypoaktive (stille), eller veksler mellom disse. Alvorlighetsgraden kan variere betydelig.

Delirium krever rask diagnostikk og intervensjon. Confusion Assessment Method (CAM) er en kort screeningtest som gir diagnosen med god presisjon (basert på DSM-III og ICD-10). Algoritmen er velegnet for påvisning og oppfølging av delirium i klinisk praksis. Spørsmålene skal besvares av helsepersonell og baseres på egen kjennskap til pasienten eller samtale med personale eller pårørende som kjenner vedkommende.

| **Dag 15**  Confusion Assessment Method (CAM) | **Sykehusskjema – IV ANTIBIOTIKA s14** |
| --- | --- |

| Inouye et al. Ann Int Med 1990; 113: 941-948.  Norsk oversettelse ved Anette Hylen Ranhoff, Marianne Hjermstad og Jon Håvard Loge, 2004. |
| --- |

**GENERELT**

Delirium (tidligere ofte kalt akutt forvirring eller akutt konfusjon) er en vanlig komplikasjon til akutt sykdom hos gamle. Det finnes flere typer, pasientene kan bli enten hyperaktive (agiterte), hypoaktive (stille), eller veksler mellom disse. Alvorlighetsgraden kan variere betydelig.

Delirium krever rask diagnostikk og intervensjon. Confusion Assessment Method (CAM) er en kort screeningtest som gir diagnosen med god presisjon (basert på DSM-III og ICD-10). Algoritmen er velegnet for påvisning og oppfølging av delirium i klinisk praksis. Spørsmålene skal besvares av helsepersonell og baseres på egen kjennskap til pasienten eller samtale med personale eller pårørende som kjenner vedkommende.

| **Dag 30** Confusion Assessment Method (CAM) | **Sykehusskjema – IV ANTIBIOTIKA s15** |
| --- | --- |

| Inouye et al. Ann Int Med 1990; 113: 941-948.  Norsk oversettelse ved Anette Hylen Ranhoff, Marianne Hjermstad og Jon Håvard Loge, 2004. |
| --- |

**GENERELT**

Delirium (tidligere ofte kalt akutt forvirring eller akutt konfusjon) er en vanlig komplikasjon til akutt sykdom hos gamle. Det finnes flere typer, pasientene kan bli enten hyperaktive (agiterte), hypoaktive (stille), eller veksler mellom disse. Alvorlighetsgraden kan variere betydelig.

Delirium krever rask diagnostikk og intervensjon. Confusion Assessment Method (CAM) er en kort screeningtest som gir diagnosen med god presisjon (basert på DSM-III og ICD-10). Algoritmen er velegnet for påvisning og oppfølging av delirium i klinisk praksis. Spørsmålene skal besvares av helsepersonell og baseres på egen kjennskap til pasienten eller samtale med personale eller pårørende som kjenner vedkommende.

| Barthel ADL-Indeks (status etter 30 dager) | **Sykehusskjema – IV ANTIBIOTIKA s16** |
| --- | --- |

| Mahoney FI, Barthel DW. Maryland State Med J 1965;14:61-65.  Denne norske versjonen er redigert i 2008 av Ingvild Saltvedt, Jorunn L. Helbostad, Unni Sveen, Pernille Thingstad, Olav Sletvold  og Torgeir Bruun Wyller på grunnlag av flere tidligere norske oversettelser og med hovedvekt på originalpublikasjonen fra 1965. |
| --- |

**GENERELT**

Barthel ADL-indeks er først og fremst beregnet på å bli brukt av sykepleiere, ergoterapeuter og fysioterapeuter i deres daglige kontakt med pasientene. Det skal registreres hva pasienten faktisk gjør, ikke hva man tror pasienten kan mestre. Svarene skal baseres på egen kjennskap til pasienten eller samtale med personale eller pårørende som kjenner vedkommende. Pasienten skal ikke ”testes”. Poengene representerer grad av uavhengighet av hjelp fra annen person, uansett årsak. Hvis det er nødvendig med *tilsyn* eller *tilrettelegging*, er personen **ikke** uavhengig, men hvis en aktivitet mestres med *hjelpemidler,* **er** personen uavhengig i denne aktiviteten.
