# Supplementary material for: A structured training program for health workers in intravenous treatment with fluids and antibiotics in nursing homes: A modified stepped-wedge cluster-randomised trial to reduce hospital admissions
Source: PLoS One. 2017 Sep 7;12(9):e0182619. doi: 10.1371/journal.pone.0182619 (PMC5589147; doi:10.1371/journal.pone.0182619)
Supplement: S5 Appendix — Form for patients treated wit iv fluids in hospital. (DOC) [file pone.0182619.s007.doc]

| Navn:________________________Født:____________ | | **Sykehusskjema – IV VÆSKE s1** | |
| --- | --- | --- | --- |
| Kjønn  K  M | |  | |
| Avdeling  Geriatrisk avdeling   Hjerteavdelingen   Infeksjonsmedisin   Hematologisk avdeling   Gastro (medisin)   Lungeseksjonen   Nyreseksjonen   Kirurgisk avdeling   Annen: _______________________ | Innleggelsestidspunkt  Dato  (ddmmåå) Kl _ _ . _ _ | |  |
| **Innleggelsesdiagnose/problemstilling** (en/flere)  1. _________________________________________  2. _________________________________________  3. _________________________________________ | **Foreløpig diagnose**(en/flere)  1. _________________________________________  2. _________________________________________  3. _________________________________________ | |  |

| **Dehydreringsårsak** | Vegrer væskeinntak  Diare  Oppkast  Feber/infeksjon  Væskende sår Medikamenter  Varmeperiode  Annet  Usikkert |  Nei  Ja   Nei  Ja   Nei  Ja   Nei  Ja ___________________________________   Nei  Ja   Nei  Ja ___________________________________   Nei  Ja   Nei  Ja ___________________________________   Nei  Ja ___________________________________ |
| --- | --- | --- |

Intravenøs væskebehandling

| Dato | Tidspunkt oppstart | Type væske | Døgndose  (ml) | Dosering | Dag  1 (x) | Dag  2 (x) | Dag  3 (x) | Dag  4 (x) | Dag  5 (x) | Dag  6 (x) | Dag  7 (x) |
| --- | --- | --- | --- | --- | --- | --- | --- | --- | --- | --- | --- |
|  |  |  |  |  |  |  |  |  |  |  |  |
|  |  |  |  |  |  |  |  |  |  |  |  |
|  |  |  |  |  |  |  |  |  |  |  |  |

Seponerte medikamenter: ____________________________________________________________________

Annen iverksatt behandling: __________________________________________________________________

_________________________________________________________________________________________________

_________________________________________________________________________________________________

Klinisk status dag 1

| BT ____/____ Puls _____ Temp ___.__ Respirasjonsfrekvens____ CRP ____  Bevissthet  Våken  Somnolent  Bevisstløs  Matinntak  Normalt  Redusert  Sonde  Væskeinntak  Normalt  Redusert  Intravenøs væske |
| --- |

|  Skjema om beslutningsprosessen fylt ut (s 4)   Confusion Assessment Method (CAM) fylt ut for dag 1 (s 5)   Kopi av medikamentliste dag 1 vedlagt | |
| --- | --- |
| Navn:________________________Født:____________ | **Sykehusskjema – IV VÆSKE s2** |

Klinisk status dag 3

| BT ____/____ Puls _____ Temp ___.__ Respirasjonsfrekvens____ CRP ____  Bevissthet  Våken  Somnolent  Bevisstløs  *Fylles ut hos de pasientene som hadde avvikende resultater dag 1* |
| --- |

|  Confusion Assessment Method (CAM) fylt ut (s 6) |
| --- |

Viktige merknader__________________________________________________________________________________

_________________________________________________________________________________________________

_________________________________________________________________________________________________

Klinisk status dag 5

| BT ____/____ Puls _____ Temp ___.__ Respirasjonsfrekvens____ CRP ____  Bevissthet  Våken  Somnolent  Bevisstløs  *Fylles ut hos de pasientene som hadde avvikende resultater dag 3* |
| --- |

|  Confusion Assessment Method (CAM) fylt ut (s 7) |
| --- |

Viktige merknader__________________________________________________________________________________

_________________________________________________________________________________________________

_________________________________________________________________________________________________

Oppsummering dag 1 etter avsluttet intravenøs væske

| **Sykdomsforløp**  ___ dager med intravenøs væske  ___ dager før klinisk frisk  Tilbake i normaltilstand?  Ja  Nei  Kjenner ikke pasientens normaltilstand   Død ___ dager etter oppstart av behandling | | |
| --- | --- | --- |
| **Komplikasjoner**  Liggesår  Fall med skade  Delir  Sykehusinfeksjon  Annet |  Nei  Ja:   Nei  Ja:   Nei  Ja   Nei Ja:  Nei  Ja: | ________________________________________  ________________________________________  ___ dager  ________________________________________  ________________________________________ |
| **Intravenøs behandling**  Komplikasjoner ved intravenøs behandling (infeksjon i venen, hematom, utstyrssvikt el.l.)  Nei  Ja:______  _________________________________________________________________________________________ | | |

| Navn:________________________Født:____________ | **Sykehusskjema – IV VÆSKE s3** |
| --- | --- |

Oppsummering 14 dager etter debut av aktuelle sykdom

|  Sykehusinfeksjon registrert (debut på sykehus/<48 t etter utskrivelse)   Nei  Ja: ____________________________________________________   Samtykke-erklæring vedlagt (pasientens underskrift på deltakelse i studien)   Barthel ADL-Indeks fylt ut på nytt - for situasjonen i dag (s 8)   Kopi av dagens medikamentliste vedlagt   Pasienten er skrevet ut til hjemmet. Ring Lisbeth Østby, 91820728   Pasienten er død ____ dager etter oppstart av behandling |
| --- |

Oppsummering ved utskrivelse (Utskrivelsesdato  ddmmåå)

| **Sykdomsforløp**  ___ dager med intravenøs væske  ___ dager før klinisk frisk  Tilbake i normaltilstand?  Ja  Nei  Kjenner ikke pasientens normaltilstand   Død ___ dager etter oppstart av behandling | | |
| --- | --- | --- |
| **Komplikasjoner**  Liggesår  Fall med skade  Delir  Sykehusinfeksjon  Annet |  Nei  Ja:  Nei  Ja:   Nei  Ja:   Nei  Ja   Nei Ja: | _______________________________________________  _______________________________________________  ___ dager  _______________________________________________  _______________________________________________ |
| **Intravenøs behandling**  Komplikasjoner ved intravenøs behandling (infeksjon i venen, hematom, utstyrssvikt el.l.)  Nei  Ja:______  _________________________________________________________________________________________  Utfordringer/ulemper ved sykehusbehandling av denne pasienten: ____________________________________  _________________________________________________________________________________________  _________________________________________________________________________________________  Fordeler med sykehusbehandling av denne pasienten: _________________________________________________________________________________________  _________________________________________________________________________________________ | | |

| Navn:________________________Født:____________ | **Sykehusskjema – IV VÆSKE s4** |
| --- | --- |

Beslutningsprosessen - Fylles ut av sykehuslege på avdelingen, helst behandlingsansvarlig lege

| 1. Var det under oppholdet noen gang tvil om denne pasienten burde vært innlagt?  Ja  Nei  Hvis ja, hva var grunnen til det (ett eller flere kryss)?   Pasienten kunne fått behandling for tilstanden på sykehjemmet   Tvil om fordelene ved innleggelse for pasienten var større enn ulempene   Tvil om livsforlengende behandling var riktig for denne pasienten   Annet _________________________________________________________________  2. Var det noen gang tvil om intravenøs behandling var riktig for denne pasienten?  Ja  Nei  Hvis ja, var det en eller flere av de følgende grunnene?   Tvil om behandlingen var til pasientens beste   Tvil om pasienten faktisk ønsket intravenøs behandling   Tvil om hva slags behandling pårørende ønsket at pasienten skulle få   Tvil om behandlingen ville gi effekt   Det var uenighet i behandlingsteamet om behandlingen   Annet ________________________________________________________________  3. Ble behandlingen diskutert med sykehjemspersonell som kjenner pasienten?  Ja  Nei  Vet ikke  Hvis nei, hvorfor ikke? ________________________________________________________________  4. Ble behandlingen på sykehuset diskutert med pasienten før oppstart?  Ja  Nei  Vet ikke  Hvis nei, hvorfor ikke? ________________________________________________________________  5. Ble behandlingen på sykehuset diskutert med pårørende før oppstart ?  Ja  Nei  Vet ikke  Hvis nei, hvorfor ikke? ________________________________________________________________  6. Ble pasientens samtykkekompetanse vurdert på sykehuset før behandlingen?  Ja  Nei  Vet ikke  Hvis ja, var pasienten samtykkekompetent?  Ja  Nei  Hvis nei, hvorfor ble samtykkekompetanse ikke vurdert (gjerne flere kryss)?   Pasienten var helt opplagt samtykkekompetent   Pasienten var helt opplagt ikke samtykkekompetent   Vi hadde ikke tid til å vurdere det   Usikkerhet i forhold til hvordan en vurderer samtykkekompetanse   Annet _________________________________________________________________  7. Er det *tidligere* (på sykehjem eller på sykehus) gjennomført samtaler med pasient eller pårørende om pasientens ønsker og verdier i forhold til livsforlengende behandling eller hva som skal gjøres ved akutt forverring av pasientens helsetilstand?  Ja  Nei  Vet ikke |
| --- |

| **Dag 1** Confusion Assessment Method (CAM) | **Skjema 3 – IV VÆSKE s5** |
| --- | --- |

| Inouye et al. Ann Int Med 1990; 113: 941-948.  Norsk oversettelse ved Anette Hylen Ranhoff, Marianne Hjermstad og Jon Håvard Loge, 2004. |
| --- |

**GENERELT**

Delirium (tidligere ofte kalt akutt forvirring eller akutt konfusjon) er en vanlig komplikasjon til akutt sykdom hos gamle. Det finnes flere typer, pasientene kan bli enten hyperaktive (agiterte), hypoaktive (stille), eller veksler mellom disse. Alvorlighetsgraden kan variere betydelig.

Delirium krever rask diagnostikk og intervensjon. Confusion Assessment Method (CAM) er en kort screeningtest som gir diagnosen med god presisjon (basert på DSM-III og ICD-10). Algoritmen er velegnet for påvisning og oppfølging av delirium i klinisk praksis. Spørsmålene skal besvares av helsepersonell og baseres på egen kjennskap til pasienten eller samtale med personale eller pårørende som kjenner vedkommende.

| **Dag 3** Confusion Assessment Method (CAM) | **Skjema 3 – IV VÆSKE s6** |
| --- | --- |

| Inouye et al. Ann Int Med 1990; 113: 941-948.  Norsk oversettelse ved Anette Hylen Ranhoff, Marianne Hjermstad og Jon Håvard Loge, 2004. |
| --- |

**GENERELT**

Delirium (tidligere ofte kalt akutt forvirring eller akutt konfusjon) er en vanlig komplikasjon til akutt sykdom hos gamle. Det finnes flere typer, pasientene kan bli enten hyperaktive (agiterte), hypoaktive (stille), eller veksler mellom disse. Alvorlighetsgraden kan variere betydelig.

Delirium krever rask diagnostikk og intervensjon. Confusion Assessment Method (CAM) er en kort screeningtest som gir diagnosen med god presisjon (basert på DSM-III og ICD-10). Algoritmen er velegnet for påvisning og oppfølging av delirium i klinisk praksis. Spørsmålene skal besvares av helsepersonell og baseres på egen kjennskap til pasienten eller samtale med personale eller pårørende som kjenner vedkommende.

| **Dag 5** Confusion Assessment Method (CAM) | **Skjema 3 – IV VÆSKE s7** |
| --- | --- |

| Inouye et al. Ann Int Med 1990; 113: 941-948.  Norsk oversettelse ved Anette Hylen Ranhoff, Marianne Hjermstad og Jon Håvard Loge, 2004. |
| --- |

**GENERELT**

Delirium (tidligere ofte kalt akutt forvirring eller akutt konfusjon) er en vanlig komplikasjon til akutt sykdom hos gamle. Det finnes flere typer, pasientene kan bli enten hyperaktive (agiterte), hypoaktive (stille), eller veksler mellom disse. Alvorlighetsgraden kan variere betydelig.

Delirium krever rask diagnostikk og intervensjon. Confusion Assessment Method (CAM) er en kort screeningtest som gir diagnosen med god presisjon (basert på DSM-III og ICD-10). Algoritmen er velegnet for påvisning og oppfølging av delirium i klinisk praksis. Spørsmålene skal besvares av helsepersonell og baseres på egen kjennskap til pasienten eller samtale med personale eller pårørende som kjenner vedkommende.

| Barthel ADL-Indeks (status etter 14 dager) | **Skjema 2 – IV VÆSKE s8** |
| --- | --- |

| Mahoney FI, Barthel DW. Maryland State Med J 1965;14:61-65.  Denne norske versjonen er redigert i 2008 av Ingvild Saltvedt, Jorunn L. Helbostad, Unni Sveen, Pernille Thingstad, Olav Sletvold  og Torgeir Bruun Wyller på grunnlag av flere tidligere norske oversettelser og med hovedvekt på originalpublikasjonen fra 1965. |
| --- |

**GENERELT**

Barthel ADL-indeks er først og fremst beregnet på å bli brukt av sykepleiere, ergoterapeuter og fysioterapeuter i deres daglige kontakt med pasientene. Det skal registreres hva pasienten faktisk gjør, ikke hva man tror pasienten kan mestre. Svarene skal baseres på egen kjennskap til pasienten eller samtale med personale eller pårørende som kjenner vedkommende. Pasienten skal ikke ”testes”. Poengene representerer grad av uavhengighet av hjelp fra annen person, uansett årsak. Hvis det er nødvendig med *tilsyn* eller *tilrettelegging*, er personen **ikke** uavhengig, men hvis en aktivitet mestres med *hjelpemidler,* **er** personen uavhengig i denne aktiviteten.
