# Supplementary material for: A structured training program for health workers in intravenous treatment with fluids and antibiotics in nursing homes: A modified stepped-wedge cluster-randomised trial to reduce hospital admissions
Source: PLoS One. 2017 Sep 7;12(9):e0182619. doi: 10.1371/journal.pone.0182619 (PMC5589147; doi:10.1371/journal.pone.0182619)
Supplement: S7 Appendix — (DOC) [file pone.0182619.s009.doc]

# Forespørsel om deltakelse i forskningsprosjektet

***”***Kan et strukturert opplæringsprogram i intravenøs behandling i sykehjem føre til

bedre pasientforløp og redusert innleggelse i sykehus?”

# Bakgrunn og hensikt

Mange eldre opplever det som en belastning å bli innlagt på sykehus. Dette er et spørsmål til deg om å gi tillatelse til at vi innhenter informasjon om deg og din akutte sykdom. Hensikten med dette er å finne ut om sykehjemsbeboere som trenger intravenøs behandling med væske eller antibiotika kan behandles like godt eller bedre på sykehjemmet som på sykehus.

# Hva innebærer studien?

Personalet ved alle sykehjem i Vestfold skal få opplæring i intravenøs behandling i løpet av de neste to årene. Dette er ikke et forskningsprosjekt, men en planlagt oppgradering av det medisinske tilbudet på hvert enkelt sykehjem. Før opplæringsprogrammet er gjennomført på ditt sykehjem, vil du og andre beboere som trenger intravenøs behandling bli innlagt på sykehus. Etter opplæringen vil de fleste kunne behandles på sykehjemmet. I noen tilfeller vil en sykehusinnleggelse fortsatt være nødvendig.

Forskningsprosjektet er en systematisk evaluering av dette opplæringsprogrammet. Vi har to hovedspørsmål vi ønsker å besvare:

1. Kan et opplæringsprogram på sykehjemmene i intravenøs behandling føre til færre sykehusinnleggelser blant sykehjemsbeboere?
2. Kan intravenøs behandling på sykehjemmet være like bra eller bedre for pasientene sammenlignet med å bli innlagt på sykehus for slik behandling?

Hver gang en sykehjemsbeboer trenger intravenøs væske eller antibiotika ønsker vi å samle inn informasjon om pasienten og sykdomsforløpet. Sykepleiere på sykehjemmet vil registrere informasjon om sykdommen og behandling som blir gitt der. For pasienter som blir innlagt på sykehus vil vi hente informasjon fra journalen om behandling, sykdomsforløp og antall dager på sykehuset.

Det er ingen ulemper eller spesielle fordeler for deg som person å gi tillatelse til at vi innhenter informasjon om deg og ditt sykdomsforløp.

**Hva skjer med informasjonen om deg?**

Informasjonen skal kun brukes i denne studien. Opplysningene vil bli behandlet konfidensielt og uten navn og fødselsnummer. Det vil ikke være mulig å kjenne igjen enkeltpersoner i resultatene av studien når disse publiseres.

**Frivillig deltakelse**Dersom du ønsker å gi oss tillatelse til å innhente informasjon om deg, undertegner du samtykkeerklæringen. Det er frivillig å delta, og du kan når som helst trekke deg uten å begrunne dette nærmere. Dersom du ikke ønsker at vi innhenter informasjon om deg, endrer det ingenting i forhold til håndteringen av din sykdom.

Dersom du har spørsmål, kan du kontakte lege og prosjektleder Maria Romøren, 91377693.

## Rett til innsyn og sletting av opplysninger om deg og sletting av prøver

Hvis du sier ja til å delta i studien, har du rett til å få innsyn i hvilke opplysninger som er registrert om deg. Dersom du trekker deg fra studien, kan du kreve å få slettet informasjonen som angår deg, med mindre opplysningene allerede er inngått i analyser eller brukt i vitenskapelige publikasjoner.

# Informasjon om utfallet av studien

Alle sykehjem i Vestfold med beboere og ansatte vil bli informert om resultatene fra studien.

Antibiotikasentret for primærmedisin ved leder Morten Lindbæk er databehandlingsansvarlig.

**Samtykke til deltakelse i studien**

Navn:

Sykehjem:

Jeg gir tillatelse til at opplysninger om meg brukes i studien

----------------- -----------------------------------------------------------------------------

(Dato) (Signatur)

Stedfortredende samtykke når berettiget, enten i tillegg til personen selv eller istedenfor

----------------- -----------------------------------------------------------------------------

(Dato) (Signatur fra pårørende)

Jeg bekrefter å ha gitt informasjon om studien

----------------- -----------------------------------------------------------------------------

(Dato) (Signatur)
