# Supplementary material for: A structured training program for health workers in intravenous treatment with fluids and antibiotics in nursing homes: A modified stepped-wedge cluster-randomised trial to reduce hospital admissions
Source: PLoS One. 2017 Sep 7;12(9):e0182619. doi: 10.1371/journal.pone.0182619 (PMC5589147; doi:10.1371/journal.pone.0182619)
Supplement: S8 Appendix — (DOCX) [file pone.0182619.s010.docx]

**The TIDieR (Template for Intervention Description and Replication) Checklist*:**


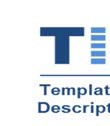

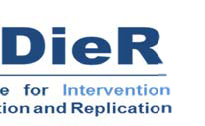


Information to include when describing an intervention and the location of the information

m

**c**

| **Item Item Where located **** | |
| --- | --- |
| **number** Primary paper  (page or appendix number) | Other † (details) |
| **BRIEF NAME**  **1.** Provide the name or a phrase that describes the intervention. Page 1  **WHY**  **2.** Describe any rationale, theory, or goal of the elements essential to the intervention. Page 4-5  **WHAT**  **3.** Materials: Describe any physical or informational materials used in the intervention, including those Page 7  provided to participants or used in intervention delivery or in training of intervention providers. Provide information on where the materials can be accessed (e.g. online appendix, URL).  **4.** Procedures: Describe each of the procedures, activities, and/or processes used in the intervention, Page 7  including any enabling or support activities.  **WHO PROVIDED**  **5.** For each category of intervention provider (e.g. psychologist, nursing assistant), describe their Page 6-7  expertise, background and any specific training given.  **HOW**  **6.** Describe the modes of delivery (e.g. face-to-face or by some other mechanism, such as internet or Page 6-7  telephone) of the intervention and whether it was provided individually or in a group.  **WHERE**  **7.** Describe the type(s) of location(s) where the intervention occurred, including any necessary Page 7  infrastructure or relevant features. | **Provided as supplemental material** |

|  | **WHEN and HOW MUCH** |  |
| --- | --- | --- |
| **8.** | Describe the number of times the intervention was delivered and over what period of time including | Page 6-7 |
|  | the number of sessions, their schedule, and their duration, intensity or dose. |  |
|  | **TAILORING** |  |
| **9.** | If the intervention was planned to be personalised, titrated or adapted, then describe what, why, | N/A |
|  | when, and how. |  |
| **10.ǂ** | **MODIFICATIONS**  If the intervention was modified during the course of the study, describe the changes (what, why, | N/A |
|  | when, and how). |  |
|  | **HOW WELL** |  |
| **11.** | Planned: If intervention adherence or fidelity was assessed, describe how and by whom, and if any | Not assessed |
|  | strategies were used to maintain or improve fidelity, describe them. |  |
| **12.ǂ** | Actual: If intervention adherence or fidelity was assessed, describe the extent to which the | Not assessed |
|  | intervention was delivered as planned. |  |

** **Authors** - use N/A if an item is not applicable for the intervention being described. **Reviewers** – use ‘?’ if information about the element is not reported/not sufficiently reported.

† If the information is not provided in the primary paper, give details of where this information is available. This may include locations such as a published protocol or other published papers (provide citation details) or a website (provide the URL).

ǂ If completing the TIDieR checklist for a protocol, these items are not relevant to the protocol and cannot be described until the study is complete.

* We strongly recommend using this checklist in conjunction with the TIDieR guide (see *BMJ* 2014;348:g1687) which contains an explanation and elaboration for each item.

* The focus of TIDieR is on reporting details of the intervention elements (and where relevant, comparison elements) of a study. Other elements and methodological features of studies are covered by other reporting statements and checklists and have not been duplicated as part of the TIDieR checklist. When a **randomised trial** is being reported, the TIDieR checklist should be used in conjunction with the CONSORT statement (see www.consort‐statement.org) as an extension of **Item 5 of the CONSORT 2010 Statement.** When a **clinical trial protocol** is being reported, the TIDieR checklist should be used in conjunction with the SPIRIT statement as an extension of **Item 11 of the SPIRIT 2013**

**Statement** (see www.spirit‐statement.org). For alternate study designs, TIDieR can be used in conjunction with the appropriate checklist for that study design (see www.equator‐network.org).
